# Transcriptome Analysis of the *Arabidopsis* Megaspore Mother Cell Uncovers the Importance of RNA Helicases for Plant Germline Development

**DOI:** 10.1371/journal.pbio.1001155

**Published:** 2011-09-20

**Authors:** Anja Schmidt, Samuel E. Wuest, Kitty Vijverberg, Célia Baroux, Daniela Kleen, Ueli Grossniklaus

**Affiliations:** 1 Institute of Plant Biology & Zürich-Basel Plant Science Center, University of Zürich, Zürich, Switzerland; 2 Smurfit Institute of Genetics, Trinity College Dublin, Dublin, Ireland; The University of North Carolina at Chapel Hill, United States of America

## Abstract

Germ line specification is a crucial step in the life cycle of all organisms. For sexual plant reproduction, the megaspore mother cell (MMC) is of crucial importance: it marks the first cell of the plant “germline” lineage that gets committed to undergo meiosis. One of the meiotic products, the functional megaspore, subsequently gives rise to the haploid, multicellular female gametophyte that harbours the female gametes. The MMC is formed by selection and differentiation of a single somatic, sub-epidermal cell in the ovule. The transcriptional network underlying MMC specification and differentiation is largely unknown. We provide the first transcriptome analysis of an MMC using the model plant *Arabidopsis thaliana* with a combination of laser-assisted microdissection and microarray hybridizations. Statistical analyses identified an over-representation of translational regulation control pathways and a significant enrichment of DEAD/DEAH-box helicases in the MMC transcriptome, paralleling important features of the animal germline. Analysis of two independent T-DNA insertion lines suggests an important role of an enriched helicase, MNEME (MEM), in MMC differentiation and the restriction of the germline fate to only one cell per ovule primordium. In heterozygous *mem* mutants, additional enlarged MMC-like cells, which sometimes initiate female gametophyte development, were observed at higher frequencies than in the wild type. This closely resembles the phenotype of mutants affected in the small RNA and DNA-methylation pathways important for epigenetic regulation. Importantly, the *mem* phenotype shows features of apospory, as female gametophytes initiate from two non-sister cells in these mutants. Moreover, in *mem* gametophytic nuclei, both higher order chromatin structure and the distribution of LIKE HETEROCHROMATIN PROTEIN1 were affected, indicating epigenetic perturbations. In summary, the MMC transcriptome sets the stage for future functional characterization as illustrated by the identification of *MEM*, a novel gene involved in the restriction of germline fate.

## Author Summary

Germline specification is a key step in sexual reproduction. In plants, the reproductive lineage or “germline” doesn't arise early in development, as it does in animals; rather, the germline is specified during flower development. In the female reproductive organs of the flower, a single sporophytic cell in each ovule is selected and differentiates into a megaspore mother cell (MMC), which will undergo meiosis. Despite the importance of the specification of the MMC as the first committed cell of the germline lineage, little is known about the genetic and molecular bases of this process. We performed a cell-type-specific transcriptome analysis of *Arabidopsis* MMCs using laser-assisted microdissection and microarrays. Statistical data analysis comparing these results with the transcriptomes of 71 other types of cells and tissues revealed the importance of translational control pathways and RNA helicases for plant germline development, a feature reminiscent of the animal germline. We further characterized a novel MMC-enriched RNA helicase, called MNEME, and showed that it plays important roles in MMC differentiation and the restriction of the plant germline to only one cell per ovule. This example illustrates the usefulness of our transcriptome dataset for the identification of novel candidate genes involved in this crucial step of plant reproduction.

## Introduction

The life cycle of flowering plants alternates between a diploid sporophytic and a haploid gametophytic phase. In contrast to animals, which form gametes directly by meitotic division from a diploid germline, plants form female and male spores by meiotic division during megasporogenesis and microsporogenesis, respectively. Subsequently, during mega- and microgametogenesis, the spores develop by mitotic division and cell differentiation into the female and male gametophytes, respectively. The two morphologically distinct gametophytes develop within specialized reproductive structures in the female and male organs of the flower, the ovules and the anthers. The multicellular, haploid gametophytes ultimately give rise to the gametes.

In the ovule, the archespore, which arises from a sub-epidermal cell, is the first cell of the reproductive lineage (“germline”) [1]. In the model plant *Arabidopsis thaliana*, the archespore differentiates directly into the megaspore mother cell (MMC), which is committed to undergo meiosis and gives rise to a tetrad of haploid megaspores. In *Arabidopsis*, as in most species, only one of these, the functional megaspore (FMS), survives while the others degenerate. The FMS occupies a defined position within the ovule suggesting that position is important for its determination and survival [2]. The importance of signaling from sporophytic ovule tissues for differentiation of the MMC and selection of the FMS has been discussed [3],[4]. The FMS develops into the haploid embryo sac (female gametophyte) through three rounds of mitosis followed by cellularization, typically forming a seven-celled embryo sac, including two gametes (the haploid egg cell and the homo-diploid central cell), two synergids, and three antipodals [2],[5],[6]. Double fertilization of the female gametes by one sperm cell each initiates seed development with the fertilized egg cell giving rise to the diploid embryo and the fertilized central cell to the triploid endosperm. Some plant species can produce asexual seeds through a process known as apomixis. To initiate apomictic reproduction, an unreduced embryo sac is formed either from a sporophytic (“somatic”) cell of the ovule (apospory) or from a MMC that omits or aborts meiosis (diplospory) [4]. The egg cell subsequently develops into an embryo without fertilization (parthenogenesis).

So far, little is known about the genes underlying the developmental program of MMC specification and differentiation. Analysis of the molecular basis underlying early reproductive development is particularly difficult due to the low abundance and inaccessibility of the relevant cells. Expression in the *Arabidopsis* MMC has so far only been shown for a few meiotic genes [7]–[11] and *SPOROCYTLESS/NOZZLE* (*SPL/NZZ*). SPL/NZZ is a plant-specific protein related to MADS-domain transcription factors, which plays an important role for the initiation of sporogenesis [12]–[15]. In *spl/nzz* mutants the nucellus is reduced and the archespore usually fails to undergo differentiation to form a MMC [12],[14]. Apart from the MMC, *SPL/NZZ* is expressed in sporophytic tissues during early stages of ovule development and in flowers, leaves, seedlings, and stems [12],[14], indicating broader functions in plant development. Interestingly, *SPL/NZZ* modulates the expression of *YUCCA2* and *YUCCA6*, genes that function in auxin biosynthesis, to regulate lateral organ development [16]. Auxin has been proposed to play an important role for gametophyte development in *Arabidopsis* [17]. It was suggested that an auxin gradient established in the developing embryo sac influences cell type specification [17].

Recently, small RNAs were shown to be involved in regulating cell fate determination by introducing epigenetic modifications at the DNA or chromatin level. ARGONAUTE (AGO) proteins are involved in this mechanism by regulating mRNAs during miRNA- or siRNA-guided post-transcriptional gene silencing. It has been demonstrated that *Arabidopsis AGO9* is required to restrict the differentiation of sub-epidermal cells into MMCs in pre-meiotic ovules [18]. In contrast to wild-type plants, more than one enlarged sub-epidermal cell was frequently observed in *ago9* mutants. Such a phenotype has so far been observed only in a small number of mutants in maize and rice [19]–[21]. In *ago9* mutants female gametophyte development from the MMC and a second sub-epidermal, sporophytic cell was observed, resembling features of apospory [18]. Enriched expression of *AGO9*, as well *AGO1*, *AGO2*, *AGO5*, and *AGO8*, was also found in the *Arabidopsis* egg cell, supporting a role of the RNA-based silencing mechanism in the female gametophyte [22]. However, a possible role of these genes in the differentiation of female reproductive structures remains to be unveiled.

To obtain more insights into the genetic and molecular bases underlying megasporogenesis in *Arabidopsis*, we used a combination of laser-assisted microdissection (LAM) and Affymetrix ATH1 GeneCHIP profiling [22] to analyze the transcriptome of the MMC and the surrounding sporophytic tissue. Statistical analyses of gene expression identified genes and functions significantly enriched in the MMC as compared to other cell types and tissues. In particular, translational regulation was identified as an important feature. Also the molecular function of ATP-dependent helicase activity was enriched. We found that mutations in a RNA-helicase gene, named *MNEME* (*MEM*) after the Greek muse of memory [23], lead to defects during megasporogenesis and mega-gametogenesis and arrest at early embryonic stages. In particular, in ovules of heterozygous *mem*/*MEM* plants, more than one enlarged sup-epidermal cell developed instead of the single MMC. Furthermore, two gametophytic cells instead of one were frequently observed, a phenotype similar to that described in *Arabidopsis ago9* mutants and mutants in the DNA-methylation pathway in maize, which affect epigenetic regulation [18],[19]. Interestingly, we observed altered epigenetic modifications in gametophytic nuclei of *mem* mutants. In summary, this study describes the transcriptional network of the *Arabidopsis* MMC suggesting a role for RNA processing and translational control at early stages of sexual reproduction and revealing an important function of a novel RNA-helicase, MEM, in the restriction of the germline lineage to only one cell per ovule.

## Results

### The *Arabidopsis* MMCs Transcriptome Encompasses over 9,000 Genes

**Figure 1:**
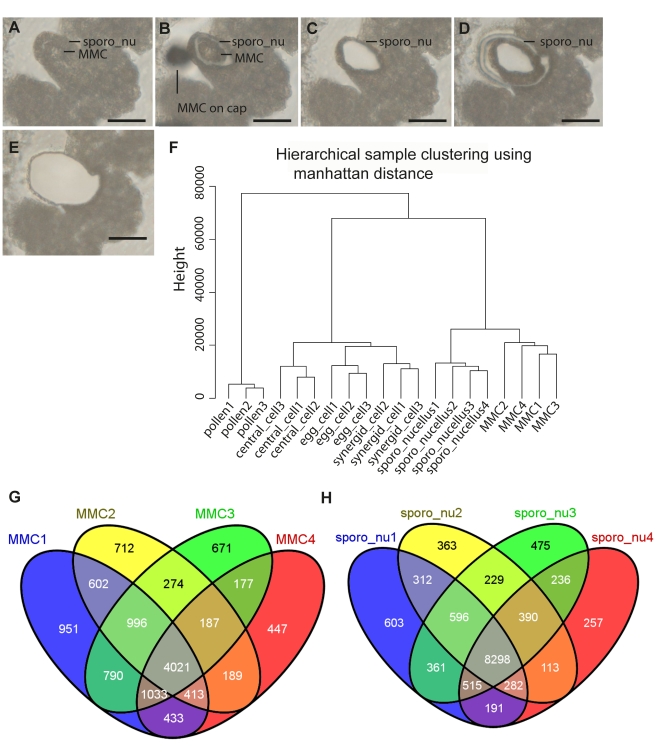
Laser-assisted microdissection (LAM) and transcriptome analysis to study megasporogenesis. (A–E) LAM of the megaspore mother cell (MMC) and the surrounding sporophytic nucellus tissue from a 6 µm dry section (scale bars 20 µm). (A) An ovule harboring the MMC before LAM. (B) The MMC was dissected from the surrounding tissue by applying the ultraviolet laser beam (diameter of ∼1 µm). Next to the nucellus is the shade of an MMC previously dissected on the surface of the isolation cap. (C) The ovule from which the MMC has been removed using an MMI isolation cap. (D–E) The surrounding sporophytic nucellus (sporo_nu) was dissected from the remaining ovule tissue (D) and removed with a separate MMI isolation cap (E). (F) Hierarchical sample clustering (manhattan distance) of female gametophytic cell types and pollen (pollen_Schmid as included in the tissue atlas [22]). Biological replicates cluster together, showing high reproducibility of the data. Samples from MMC (megaspore_mothercell) and the surrounding nucellus tissue (sporo_nucellus, sporo_nu) form a close cluster when compared to the mature female and male gametophyte, indicating their close relationship in terms of cell lineage. (G–H) Venn diagrams showing the overlaps of predictions of gene expression (present calls) as determined with the *At*PANP algorithm.

To investigate the transcriptome of the *Arabidopsis* MMC and the surrounding sporophytic tissue of the ovule (sporo_nucellus), we combined LAM with microarrays (Figure 1). MMCs and the surrounding nucellar tissue were isolated separately by LAM (Figure 1A–E). Because of the small size of the ovules at this young developmental stage and the structural limitations of dried sections required for LAM, limited cross-contamination of the samples could not completely be avoided. Between 560 and 930 sections were pooled per sample. The extracted total RNA was subjected to two rounds of linear amplifications, labeled, and hybridized to Affymetrix ATH1 arrays. As the default algorithm for the generation of present and absent calls performs poorly on data from amplified samples [22],[24], an alternative algorithm, *At*PANP, was adapted and applied to calculate present/absent *p* values [22]. This algorithm has been shown to outperform the default algorithm for the generation of present and absent calls in terms of accuracy and precision on data from cell-type-specific LAM samples [22]. However, the *At*PANP algorithm was based on non-matching probes on the ATH1-array to determine the background signal in accordance with *Arabidopsis* TAIR7 genome annotation [22]. Therefore, we updated the array annotation and the negative probe selection based on the TAIR9 genome release (http://www.arabidopsis.org; http://brainarray.mbni.med.umich.edu/Brainarray/default.asp). The re-annotated array targets 21,504 genes, which is 75% of all genes annotated in the genome (64% including putative pseudogenes and transposable element genes). A total of 6,650 genes were found to be expressed in the MMC (i.e., significantly detected above background, hereafter referred to as present/P) in three out of four arrays (Figure 1G), and an additional 2,465 genes were detected as present in two MMC replicates (referred to as marginal/M; Table S1). Together, 9,115 genes showed putative expression in the MMC, which is a bit more than the 8,850 genes identified to be expressed in the mature female gametophyte [22]. For the sporophytic nucellar tissue, 10,081 genes were detected as P, while an additional 1,442 genes were M (Figure 1H). This is in agreement with the expectation that more genes are expressed in the heterogeneous nucellus tissue as compared to a single cell type.

### Data Validation Indicates High Accuracy of the MMC Dataset

**Figure 2:**
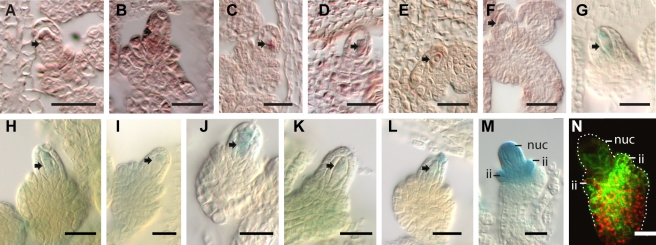
Independent data validation. Data validation for genes preferentially expressed in the MMC (A–L) or expressed in the nucellus tissues (M–N). Scale bars are 20 µm, arrows point towards the MMC, ii (inner integuments), nuc (nucellus). (A–F) In situ hybridizations with antisense probes for *AT3G14700*, encoding a SART-1 family protein (A) *AT2G30940*, a gene encoding a protein kinase (B), the transcription factor gene *AT1G31150* (C), *AT2G29210* coding for a splicing factor PWI domain-containing protein (D), *PUMILIO23* (*PUM23*, *AT1G72320*) (E), and *AT5G23080* encoding TOUGH (TGH), which interacts with TATA-box binding protein 2 (F). (G–J) GUS staining of plant lines expressing translational fusions of the putative promoter regions of *AT1G11270* encoding an F-box and associated interaction domains containing protein (G), *AT3G19510* encoding HAT3.1 that belongs to the family of PHD-finger homeodomain proteins (I), *AT3G21175* encoding GATA transcription factor 24 (H), and *AT2G24500* coding for the C2H2 zinc finger protein FZF (J) with the *E. coli uidA* gene. (K, L) GUS staining of ET4022 and ET7943 with the enhancer trap element inserted in the genomic regions of *AT1G31240* and *AT1G80440*, encoding a bromodomain transcription factor and a galactose oxidase/kelch repeat superfamily protein, respectively. (M) GUS staining with a line expressing the *E. coli uidA* gene under control of the *PUM12* promoter [22]. (N) GFP signal observed in lines carrying the *pABCB19:ABCB19-GFP* construct reporting expression of an ATP-binding cassette (ABC) transporter [25].

We validated our dataset using different independent approaches: (I) expression analysis using RNA in situ hybridization, (II) analysis of reporter gene expression in transgenic plant lines carrying putative *cis*-regulatory elements driving the *E. coli uidA* gene encoding ß-glucuronidase (GUS), (III) investigation of enhancer trap lines, and (IV) comparison to the literature. For 12 genes we could confirm predominant or exclusive expression in the MMC within developing ovules (Figure 2A–L, Table S2). In addition, *PUMILIO12* (*PUM12*) and *ATP-BINDING CASSETTE B19* (*ABCB19*) were confirmed to be expressed in the nucellus tissue, using lines expressing *GUS* and *GFP* as reporters, respectively (Figure 2M and 2N) [22],[25]. To date only five genes have been described to be expressed in the MMC: the meiotic genes *DISRUPTION OF MEIOTIC CONTROL1* (*DMC1*), *SOLO DANCERS* (*SDS*), *DYAD/SWITCH1* (*SWI1*), *MULTIPOLAR SPINDLE1* (*MPS1*), and *SPL/NZZ* [7]–[12],[14]. *DMC1*, *SDS*, and *DYAD/SWI* are important for homologous recombination, sister chromatid cohesion, synapsis, and bivalent formation during meiotic prophase I, and *MPS1* is a gene involved in spindle organization in meiocytes [7]–[11],[26]. Except for *MPS1* these genes were present in our MMC dataset; *SWI1* could not be analyzed as it is not represented on the ATH1 array. In addition, *NZZ/SPL*, *WUSCHEL* (*WUS*), *WINDHOSE1* (*WIH1*), *WIH2*, and *AGO9* were present in the dataset from surrounding nucellus tissue, consistent with the literature [12]–[15],[18],[27],[28]. These independent validations provide strong evidence for the accuracy of the expression datasets.

The MMC undergoes meiosis and eventually gives rise to the haploid embryo sac. Consequently, genes encoding proteins important for meiosis are expected to be expressed in this cell type. However, although mainly developmental stages starting just before meiosis and ending at prophase of meiosis I were sampled, it should be noted that development from archesporial cell to mature MMC is a continuous process, such that the samples contain multiple developmental stages. We found that five additional genes active during female meiosis without known functions in somatic tissues were expressed in the MMC (P or M). *MUTL-HOMOLOGUE1* (*MLH1*), *MUTS HOMOLOG4* (*MSH4*), *RECOMBINATION8*/*SYNAPTIC1* (*REC8*/*SYN1*), and *PARTING DANCERS1* (*PTD1*) function during prophase of meiosis I, and *TARDY ASYNCHRONOUS MEIOSIS* (*TAM*) controls the transitions between prophase and the first meiotic division as well as between meiosis I and meiosis II [29]–[35]. The identification of genes with important roles during prophase of meiosis I as expressed in our MMC dataset is consistent with the developmental stages covered in our sampling.

### Analysis of Gene Expression and Gene Ontologies Enriched in the MMC Reveal Translational Control Pathways and RNA-Helicases as Distinctive Features

To identify new genes with a role in MMC specification and differentiation and to obtain novel insights into the transcriptional basis and molecular mechanisms underlying megasporogenesis, we analyzed our transcriptome datasets of the MMC and the surrounding sporophytic nucellus tissues by hierarchical clustering and an analysis of gene enrichment. In particular we compared the transcriptome of the MMC with the transcriptomes of (I) the surrounding nucellar tissue, (II) the cells of the mature female gametophyte, and (III) an additional 70 gametophytic and sporophytic cell types and tissues from a tissue atlas (as described in [22] plus additional samples, see Methods). In addition, we compared (IV) the expression in the nucellar tissue with the tissue atlas.

The MMC develops from the selected archespore, which is closely related in cell lineage to the surrounding tissue. It can thus be assumed that they share, to a certain extent, similar gene expression patterns. Nevertheless, the MMC is morphologically and functionally distinct from the surrounding cells. The determination of the MMC can be viewed as the delineation of a committed cell lineage that corresponds to the animal germline. Thus, the MMC and the egg cell of the mature embryo sac are the first and the last stage of the plant germline lineage. To relate the transcriptome of the MMC and surrounding tissue to the recently investigated transcriptomes of cell types of the mature female gametophyte (egg cell, central cell, and synergids) and to the male gametophyte (pollen), we applied hierarchical agglomerative sample clustering. Cell-type- and tissue-specific datasets cluster together, indicating good reproducibility of the data (Figure 1F). All datasets from the female germline lineage and the sporophytic nucellus tissue cluster closer together and group separately from pollen. In addition, the MMC shares more characteristics with the sporophytic nucellar tissue than with gametophytic cells, in agreement with their close relationship with respect to cell lineage.

**Figure 3:**
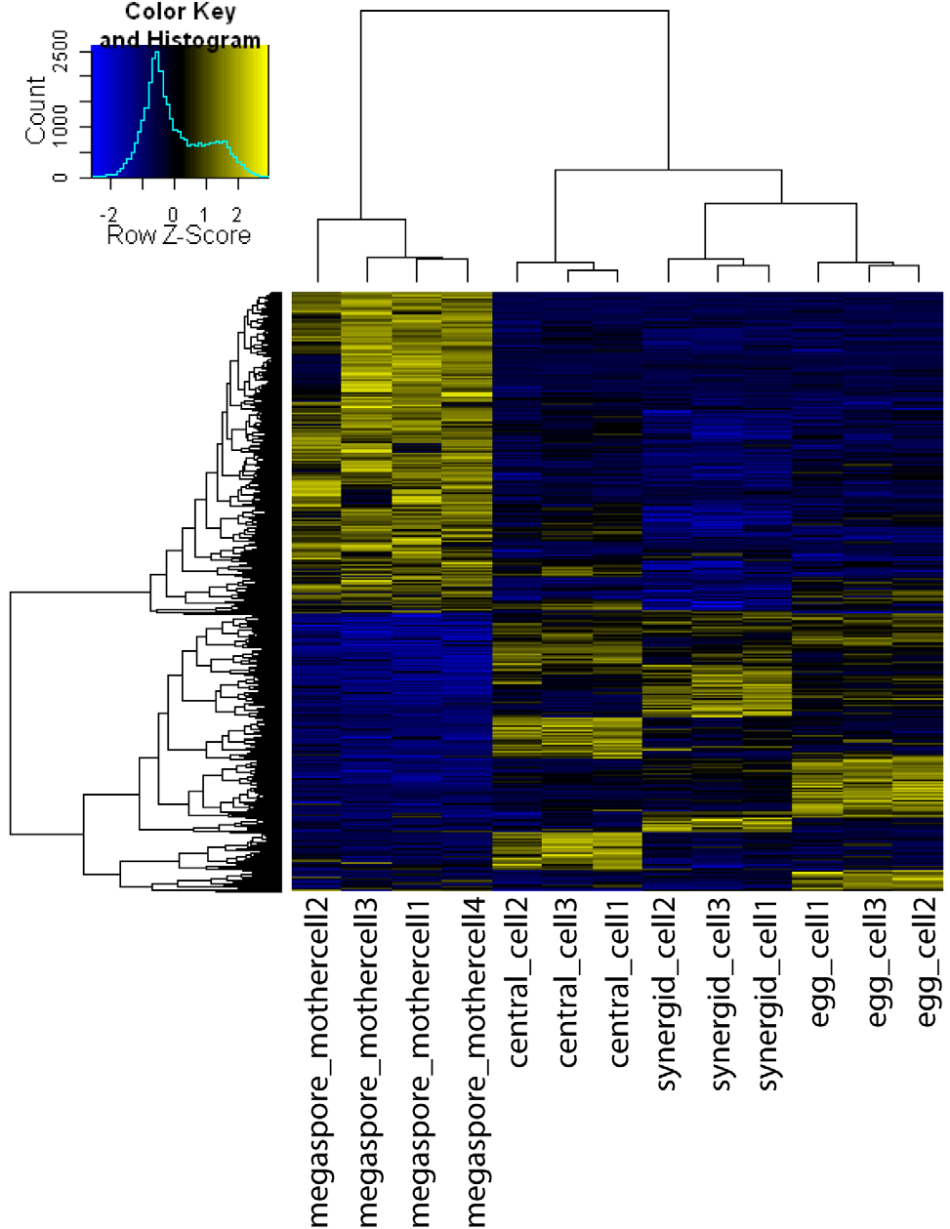
Heatmap of expression values for genes differentially expressed in MMCs and the mature female gametophyte. Heatmap of log2-scale expression values for genes significantly differentially expressed in MMC (megaspore_mothercell) and the cells of the mature female gametophyte (egg cell, central cell, synergids). Hierarchical clustering of genes/samples was based on euclidean distance and hierarchical agglomerative clustering. Colors are scaled per row and yellow denotes high expression and blue low expression.

The mature female gametophyte is separated from the MMC by only a few cell cycles. Potentially, they share expression of a subset of genes important for the identity of the germline lineage. However, other genes will be important either for differentiation of the female gametophyte and the gametes or for MMC specification and megasporogenesis, the transition from the sporophytic to the gametophytic phase. Thus, a comparison of transcriptional profiles from these two developmental stages can provide important insights into the molecular basis of cell specification and cell fate acquisition. We found 2,451 genes differentially expressed in the MMC and the cells of the mature female gametophyte (egg cell, central cell, and synergid cells [22]) at a false discovery rate below 0.05 (Figure 3) [36]. We now focused on 796 genes with significantly enriched expression in the MMC in all three contrasts. A functional classification of these genes identified translational regulation control pathways and functions related to ribosome biogenesis and structure as highly over-represented (*p* value <0.01, Table 1, Table S3). In addition, mainly different metabolic functions and transport processes, particularly for the transport of different ions, were significantly enriched (*p* value <0.01, Table 1, Table S3), but also the molecular functions “structural constituent of chromatin” and “ATP-dependent helicase activity” (Table S3). Interestingly, genes annotated in the gene ontology (GO) term “embryonic development” also were identified as near significantly enriched (*p* value  = 0.011, Table 1, Figure S1).

**Table 1:** Gene ontology analysis.

GO.ID	Term	Significant	Expected	*p* Value
GO:0006412	translation	91	17.8	<1e-30
GO:0042254	ribosome biogenesis	29	4.74	6.6e-14
GO:0010038	response to metal ion	37	12.49	9.7e-09
GO:0006970	response to osmotic stress	33	13.52	3.5e-06
GO:0009067	aspartate family amino acid biosynthetic process	8	1.01	4.3e-06
GO:0006096	glycolysis	8	1.71	0.0003
GO:0009409	response to cold	20	8.39	0.0003
GO:0015986	ATP synthesis coupled proton transport	5	0.70	0.0005
GO:0006839	mitochondrial transport	9	2.41	0.0006
GO:0006511	ubiquitin dependent protein catabolic process	18	7.73	0.0008
GO:0006457	protein folding	18	8.27	0.0017
GO:0015691	cadmium ion transport	3	0.31	0.0028
GO:0006094	gluconeogenesis	3	0.35	0.0042
GO:0000060	protein import into nucleus, translocation	2	0.12	0.0044
GO:0042732	D-xylose metabolic process	2	0.12	0.0044
GO:0015692	lead ion transport	2	0.16	0.0086
GO:0006556	S-adenosylmethionine biosynthetic process	2	0.16	0.0086
GO:0006633	fatty acid biosynthetic process	9	3.57	0.0094
GO:0009790	embryonic development	23	13.75	0.011

Biological processes identified to be upregulated based on the 796 genes enriched in the MMC transcriptome as compared to the transcriptomes of egg cell, central cell, and synergids (*p* value <0.01 regarded as significant, *p* value 0.011 as near significant).

To obtain more insight into the molecular mechanisms underlying the development of the MMC in contrast to the mature gametophyte, we analyzed our dataset for enrichment of protein family (PFAM) domains and gene families. Three gene families, the “cytoplasmic ribosomal gene family”, the “eukaryotic initiation factor family”, and the “proton pump interactor (PPI)” gene family, as well as 34 PFAM domains, including 10 ribosomal protein domains, were identified as significantly enriched (Table S4, Fisher's exact test, *p* value <0.01). In addition, the “HMG (high mobility group) box”, the “eIF-6 family”, and the “DEAD/DEAH-box helicases” belonged to the protein domains significantly over-represented (Table S4). Together, this analysis suggests that translational regulation is a major feature underlying MMC specification, paralleling an important feature of the animal germline (reviewed in [37]). In addition, specific RNA-helicases play crucial roles in germline development in animals (reviewed in [37]). Interestingly, DEAD/DEAH-box helicases were specifically enriched in the MMC as compared to the cells of the embryo sac. With few exceptions, these genes were also more highly expressed in the MMC than in pollen or sperm (Figure S2), supporting their importance for megasporogenesis as compared to gamete differentiation.

**Figure 4:**
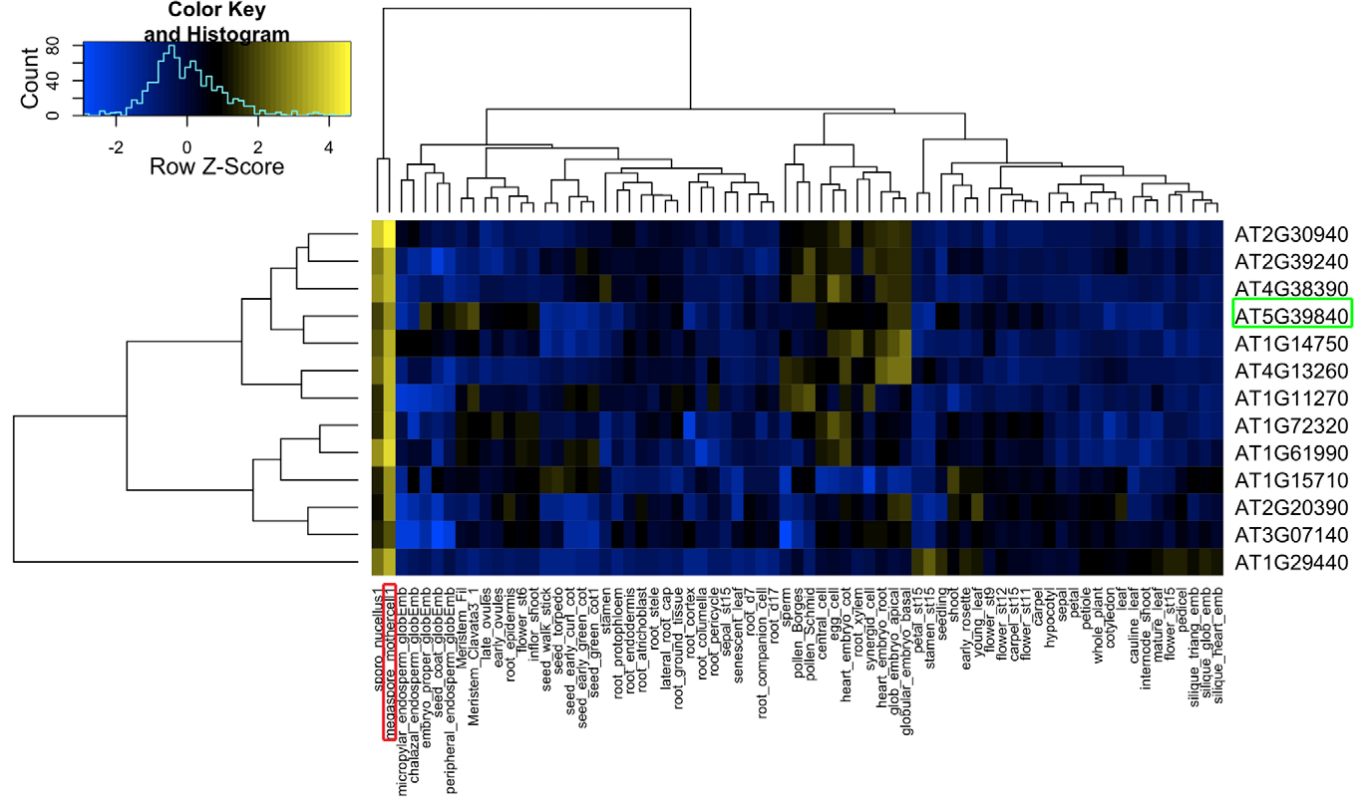
Heatmap of expression values for genes enriched in MMCs as compared to the tissue atlas. Heatmap of log2-transformed mean expression values showing 13 genes significantly enriched in the MMC samples as compared to the tissue atlas composed of a total of 72 from different *Arabidopsis* cell types and tissues (*p* value <0.01 after Benjamini-Hochberg adjustment). Hierarchical clustering of genes/samples was based on euclidean distance and hierarchical agglomerative clustering. Colors are scaled per row and yellow denotes high expression and blue low expression. Red box, MMC; green box, *MEM*.

The comprehensive tissue atlas allowed us to identify genes with preferential expression in the MMC and the surrounding nucellus tissue. In the nucellus tissue 134 genes were significantly enriched as compared to the tissue atlas not including the MMC (adjusted *p* value <0.01 [38], Table S5). Functional gene classification identified the molecular functions “acid phosphatase activity”, “protein serine/threonine phosphatase activity”, “structural constituent of ribosome”, “RNA binding”, and the biological process “oligopeptide transport” as upregulated in nucellus tissue (Table S6). One of the oligopeptide transporters significantly enriched in the nucellus, *OLIGOPEPTIDE TRANSPORTER9* (*OPT9*), was previously described as highly expressed in microspores and bicellular pollen [39], suggesting a role during reproductive development. Including the MMC in the analysis, 49 genes were significantly enriched in nucellus tissue as compared to the tissue atlas (Figure S3, adjusted *p* value <0.01 [38]). Analysis of this set of genes revealed the gene families “cytochrome P450” and “monolignol biosynthesis” as significantly enriched (Fisher's exact test, *p*-value <0.01). In the MMC, 82 genes were significantly enriched as compared to the tissue atlas (excluding sporo_nucellus, Figure S4, Table S7, adjusted *p* value <0.01 [38]). Based on these genes, functional gene classification suggests roles for “tyrosine biosynthesis”, “translation”, “acid phosphatase activity”, and “ATP-dependent RNA-helicase activity” for MMC differentiation (Table S8). When including the nucellus samples in the tissue atlas, still 13 genes were significantly enriched in the MMC, suggesting that those genes might play specific roles during MMC specification and differentiation (Figure 4).

Among these 13 genes is *SDS,* involved in homologous chromosome pairing during meiotic prophase I [8]. *AT2G20390* and *AT4G38390* encode unknown proteins. *AT3G07140* encodes a GPI-transamidase GPI16 subunit protein, with a putative function in adding GPI anchors to proteins linked to the cell surface. *AT2G30940* (Figure 2B), encoding a protein tyrosine kinase, and *AT1G11270* (Figure 2G), coding for a Cyclin-like F-box protein, are enriched in the MMC, potentially with functions in inter- or intra-cellular signaling and cell cycle regulation, respectively. *AT2G39240* encodes an RNA polymerase I transcription factor, and *AT1G61990* encodes a protein related to mitochondrial transcription factors. *Arabidopsis* PUMILIO23 (AtPUM23*)* is an RNA-binding protein located in the nucleus [40]. *AT1G15710* is a prehenate dehydrogenase potentially involved in tyrosin biosynthesis. Also *YUCCA2*, a gene involved in auxin biosynthesis and *AT1G29440*, encoding an auxin-responsive gene related to *SMALL AUXIN UPREGULATED68* (*SAUR68*), are predominantly expressed in the MMC, supporting the importance of auxin signaling for early stages of reproductive development. In addition, an ATP-dependent RNA-helicase, *AT5G39840*, which we named *MNEME* (*MEM*) after the Greek muse of memory [23], is amongst these 13 genes specifically enriched in the MMC. Although only two of the genes are annotated as unknown proteins, none of these genes have been functionally characterized in detail so far, except for *YUCCA2* and *SDS*. This might be due to their rather specific expression in a rare cell type. Interestingly, we discovered the expression of DEAD/DEAH-box helicases as well as genes with functions related to translation also in the comparison of the MMC transcriptome against the tissue atlas, supporting the evidence that these are dominant features of the MMC.

### The MEM RNA-Helicase Controls Germline Specification and Is Required for Embryo Sac and Seed Development

Our transcriptional dataset suggests the importance of DEAD/DEAH-box helicases during early developmental stages of the female reproductive lineage. One of the helicases, *MEM*, is encoded by one of the genes preferentially expressed in the MMC (Figure 4, Figure S5, Table S9), suggesting for a role in MMC specification and differentiation. To study the potential function of *MEM* during reproductive development, we analyzed two independent T-DNA insertion lines, *mem-1* and *mem-2*, inserted in the first exon and in the 3′UTR 50 bp downstream of the stop codon, respectively. Indeed, heterozygous *mem-1* and *mem-2* plants showed fertility defects with 40% (*N* = 563) and 33% (*N* = 627) of arrested ovules or aborted seeds, respectively. Transmission efficiency of the mutant alleles was analyzed in reciprocal crosses of heterozygous *mem-1/MEM* or *mem-2/MEM* plants with the wild type and showed a reduced transmission through the female but not the male gametophyte (Table 2, [41]). This indicates that *mem* is a female gametophytic mutant. Indeed, only 4% (*N* = 214) of seeds were arrested or developmentally delayed after pollinating wild-type flowers with pollen of *mem-2/MEM* plants, in contrast to 26% (*N* = 142) of arrested seeds observed in siliques of heterozygous *mem-2/MEM* plants pollinated with wild-type pollen. Although *mem* is transmitted through both male and female gametophytes, homozygous plants have not been identified, indicating that they are either not viable or only survive at very low frequency, implying embryo lethality.

**Table 2:** Transmission efficiencies (TE) of *mem-1* and *mem-2* alleles.

Cross	Number Heterozygous Observed	Number WT Observed	Number Heterozygous Expected	Number WT Expected	Total	X^2^	Transmission Efficiency (TE) in %
*mem-1*/*MEM* × WT	16	133	74.5	74.5	149	91.9	TE_F_ = 12.0
WT × *mem-1*/*MEM*	75	78	76.5	76.5	153	0.5	TE_M_ = 96.2
*mem-2*/*MEM* × WT	32	109	70.5	70.5	141	42.1	TE_F_ = 29.3
WT × *mem-2/MEM*	78	54	66.0	66.0	132	4.4	TE_M_ = 144.4

Analysis of transmission efficiencies (TE) by PCR based genotyping of heterozygous *mem-1/MEM* and *mem-2/MEM* mutants reciprocally crossed to wild-type (WT). X^2^<χ^2^_0.05_ = 3.85, [41] corresponds to a significant deviation from the expected segregation rate. TEs are significantly reduced when inherited via the female, but not the male parent.

**Figure 5:**
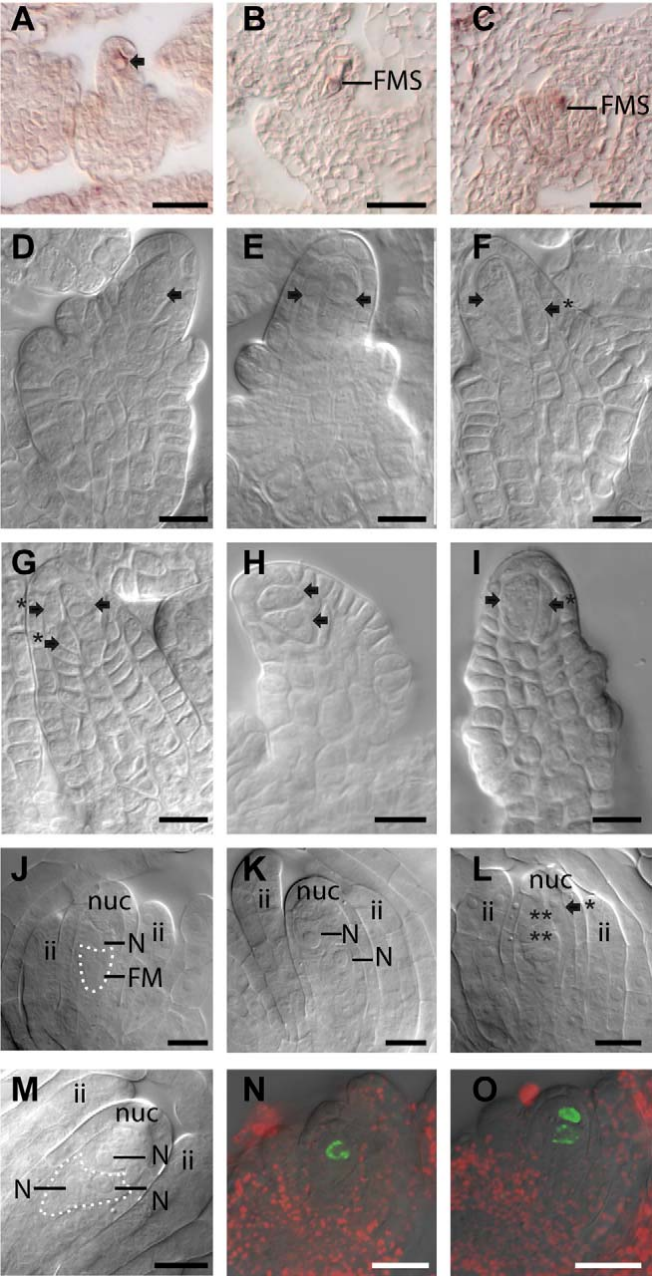
Analysis of *MEM* expression and *mem-1* and *mem-2* mutant phenotypes during megasporogenesis. (A–C) In situ hybridization showing expression of *MEM* in the MMC (A), the degenerating tetrad (B), and the functional megaspore (B, C) in wild-type plants using an antisense probe targeting *MEM*. (D–I) Differentiation of MMCs in ovules of heterozygous *mem-1/MEM* (D, H, I) and *mem-2/MEM* (E, F, G) mutant plants. In developing ovules, either one normally differentiated MMC (D), two MMCs (E, H), or an MMC with one or more smaller unusual adjacent cells were observed (F, G, I). (J–M) Two gametophytic cells with FMS characteristics were often observed at the FMS stage (J, K) or after the first mitotic division (M), or abnormal cells were seen adjacent to the degenerated megaspores (L) in ovules of *mem-1/MEM* (J) and *mem-2/MEM* (K–M) mutant plants. (N, O) Analysis of H2B-YFP expression under the control of the *AKV* promoter in plants heterozygous for the *mem-2* allele. YFP signals were studied by confocal microscopy. The *AKV* cell identity marker indicates one gametophytic cell in the wild-type ovule (N) and two gametopyhtic cells in the mutant ovule (O) at the FMS developmental stage. Scale bars are 20 µm (A–C, N, O) and 10 µm (D–M) (arrows point to MMC, an arrow with an asterisk indicates abnormal adjacent cell, two asterisks denote degenerated megaspores, ii inner integuments; nuc, nucellus; N, nucleus).

As *MEM* is predominantly expressed during early stages of reproduction, we first studied megasporogenesis in plants carrying a mutant *mem-1* or *mem-2* allele in more detail. In wild-type plants, one archespore becomes selected in the sub-epidermal layer of the ovule and differentiates into a MMC. However, in 6% (*N* = 141) of wild-type ovules, we observed initiation of two MMCs before meiosis, in agreement with the 5%–6% reported previously [2],[18]. In ovules of *mem-1/MEM* and *mem-2/MEM* plants, however, 18% (*N* = 275) and 22% (*N* = 171) form either more than one enlarged sub-epidermal cell with characteristics of the MMC or an MMC with adjacent abnormal cells (Figure 5E–I).

In addition, at the onset of megagametogenesis, instead of one FMS and the remnants of the three degenerated megaspores, a second gametophytic cell was often observed, or the FMS was flanked by abnormal cells (Figure 5J–L). To analyze whether these cells are differentiated gametophytic cells, we used the *ANTIKEVORKIAN* (*AKV*) cell-identity reporter previously shown to mark nuclei during megagametogenesis prior to cellularization [42],[43]. In wild-type plants, this reporter was expressed in the FMS, but not in the degenerated megaspores (Figure 5N). Occasionally, a very weak staining was observed in the degenerated megaspore adjacent to the differentiating functional megaspore (∼10%, *N* = 87). In heterozygous *mem-1* or *mem-2* mutant plants, however, nuclei of two adjacent cells were often marked as gametophytic (Figure 5O), as observed in ∼34% (*N* = 134) and ∼33% (*N* = 123) of analyzed ovules, respectively. An increased number of gametophytic nuclei were often observed during early stages of megagametogenesis, likely derived from two female gametophytes (Figure 5M, Figure S6I–K). In addition, the shape of the gametophyte (Figure S6A and S6B) and the positioning of gametophytic cells in the ovule, or nuclei in the gametophyte, were affected (Figure S6B–D,H,L). Therefore, a second gametophytic cell likely initiated gametophyte development resulting in an unusually positioned developing embryo sac (Figure S6C and S6D).

**Figure 6:**
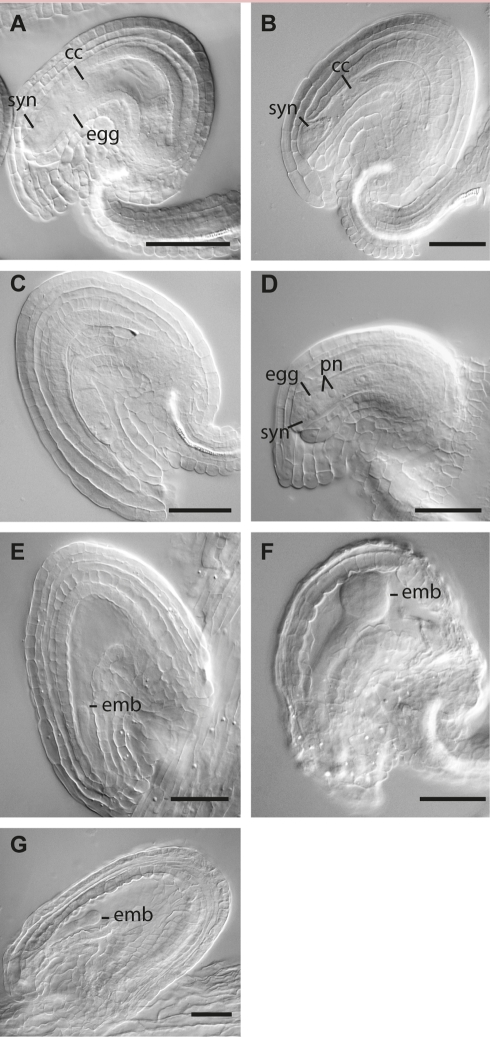
Phenotypic analysis of *mem-1* and *mem-2* mature gametophytes and early embryogenesis. (A) Wild-type embryo sac. (B–D) Typical phenotypes observed in mature female gametophytes of plants heterozygous for *mem-1*. Abnormally narrow shaped embryo sacs (B), ovules with differentiated sporophyte but without discernible gametophyte (C), and gametophytes with unfused polar nuclei (pn) were observed (D). (E–G) Arrest of embryonic development in mutants heterozygous for *mem-1* (E, F) or *mem-2.* (F) Embryo developmental arrest was typically observed latest at globular stage. (A–G) Scale bars are 40 µm; egg, egg cell; syn, synergids; cc, central cell; emb, embryo; pn, polar nuclei.

To determine whether the two FMS-like cells give rise to two mature embryo sacs in one ovule and whether megagametogenesis in mutant ovules could give rise to normally developed mature gametopyhtes, flowers of heterozygous *mem-1/MEM* and *mem-2/MEM* mutant plants were analyzed 3 days after emasculation. Although a second normal mature embryo sac was never observed, in *mem-1/MEM* at least 44% (*N* = 177) showed mutant phenotypes in the mature female gametophyte. An additional 8% of all ovules could not clearly be classified. In the most abundant mutant class, the female gametophyte harbored a normal structure with all cell types except that the polar nuclei in the central cell did not fuse (23% of total ovules analyzed, Figure 6D). In the second most abundant mutant class, gametophytes were abnormally narrow with fused polar nuclei (13%) (Figure 6B). Other phenotypes included untypical positioning of the putative egg cell or other cells (∼3%) and absent gametophytes (∼5%, Figure 6C). Similar phenotypes were observed in ovules of *mem-2/MEM* plants (Figure S6E–G). In summary, heterozygous plants carrying a mutant *mem-1* or *mem-2* allele (I) are affected during megasporogenesis, particularly in the selection of the MMC and FMS, indicating haplo-insuffiency of the *MEM* gene, and (II) have a gametophytically controlled defect in the development of the embryo sac and seed.

### *MEM* Is Required to Establish the Proper Epigenetic State of Gametophytic Nuclei

Double fertilization initiated seed development but, in comparison to the wild type, developmental progression was delayed in *mem-1* and *mem-2* derived seeds, which finally arrested at different early embryonic stages (from one-cell to mid-globular stage, Figure 6E–G, Figure S7). To gain more insights into the embryonic function of *MEM* we studied embryogenesis in *mem-1/MEM* mutant plants in more detail. At 2 days after pollination (DAP), when the majority of wild-type embryos had undergone two or three cell divisions (two- to four-cell embryo proper), the majority of mutant embryos had divided only once or not at all (Figure S7A and S7C–E). Endosperm development was delayed in comparison to the wild type (Figure S7C–E). At 3 DAP, a proportion of unfertilized ovules and seeds (likely arrested around the zygote stage) had started degeneration and collapsed (Figure 6E, Figure S7B and S7F). Only about 10% of embryos with a developmental delay developed into a two- or four-cell embryo, while the majority of wild-type embryos had reached the octant or early globular stage (Figure S7B). At 4 DAP, the majority of arrested seeds had collapsed and only infrequently, in about 1% of all ovules and seeds (*N* = 149), arrest at the mid-globular stage was observed (Figure 6G).

As the *MEM* gene was identified as significantly enriched in the MMC, this finding suggests that either (I) carryover of stable transcripts present in the MMC of a heterozygous plant is enough to sustain later stages until early seed development, (II) transcripts present in the selected MMC prevent early arrest, but *de novo* transcription is required at later developmental stages, or (III) during early stages of reproduction *MEM* determines the developmental fate of the gametophyte (e.g., by setting an epigenetic state that is interpreted only later in development). Alternatively, (IV) other ATP-dependent RNA-helicases enriched in the MMC might act redundantly during megasporogenesis.

As a first approach to investigate these possibilities, we studied the transcript abundance and expression during reproductive development in more detail. By array analysis of the cells of the mature female gametophyte [22], expression of *MEM* was neither observed in the gametes (egg, central cell, sperm) or the synergids (Figure 4, Table S9), nor in the transcriptomes of embryo and endosperm, except for marginal expression in one globular embryo sample (embryo_proper_globEmb; P in one of two replicates; Table S9). To confirm the transcriptome data we analyzed the expression of *MEM* by RNA in situ hybridization during megasporogenesis on buds harboring mature female gametophytes, and during early seed development. Highest expression was detected during megasporogenesis, in the archespore (Figure S5A), the MMC (Figure 5A, Figure S5B), and the FMS (Figure 5B and 5C). Weaker signals were detected in the sporophytic tissues of the developing ovule, while no specific signals were detected in the mature gametophyte or the sense controls (unpublished data and, Figure S5C). These data independently confirm the accuracy of our transcriptome dataset and show that *MEM* is highly expressed in the MMC and FMS, while it is either absent or strongly down-regulated in the mature female gametophyte. During early stages of seed development, a weak signal was detected in the endosperm, while in embryos signals were rarely observed and hardly distinguishable from background, likely due to very low transcript levels at the detection limit (Figure S5D–F).

The specific enrichment of *MEM* expression during megasporogenesis together with the developmental arrests of the embryo sac or early embryo suggested that *MEM* might either directly or indirectly determine molecular responses that occur later in development. In plants as well as in animals, epigenetic modifications based on histone modifications and DNA-methylation play important roles in regulating gene expression. Such epigenetic marks determine the chromatin structure and, thus, the transcriptional state of a cell (reviewed by [44],[45]). The LIKE HETEROCHROMATIN PROTEIN1/TERMINAL FLOWER2 (LHP1/TFL2) protein has previously been shown to associate with euchromatic repressive marks [42],[46]. It binds to H3K27me3 methylation marks established by *Polycomb* group proteins in euchromatic regions and is of functional importance for the interpretation of these marks [47]–[49]. In the *Arabidopsis* mature female gametophyte, LHP1 binds repressive chromatin marks in the nuclei of the egg cell and the synergids and, to a much lower extent, the central cell [42]. In the mature gametophyte before fusion of the polar nuclei it is equally expressed in the two unfused polar nuclei and the egg cell and synergid nuclei [42]. To study potential changes in the establishment of this epigenetic mark in gametophytes of heterozygous *mem-1* and *mem-2* mutant plants, we analyzed the distribution of LHP1 in mature embryo sacs. We crossed plants carrying a LHP1/TFL2 construct in translational fusion to GFP (*pTFL2:TFL2-GFP*; [46]) to heterozygous *mem-1/MEM* and *mem-2/MEM* plants. Plants of the F2 generation from these crosses were selected for the *TFL2-GFP* marker and the presence of the *mem-1* or *mem-2* allele.

**Figure 7:**
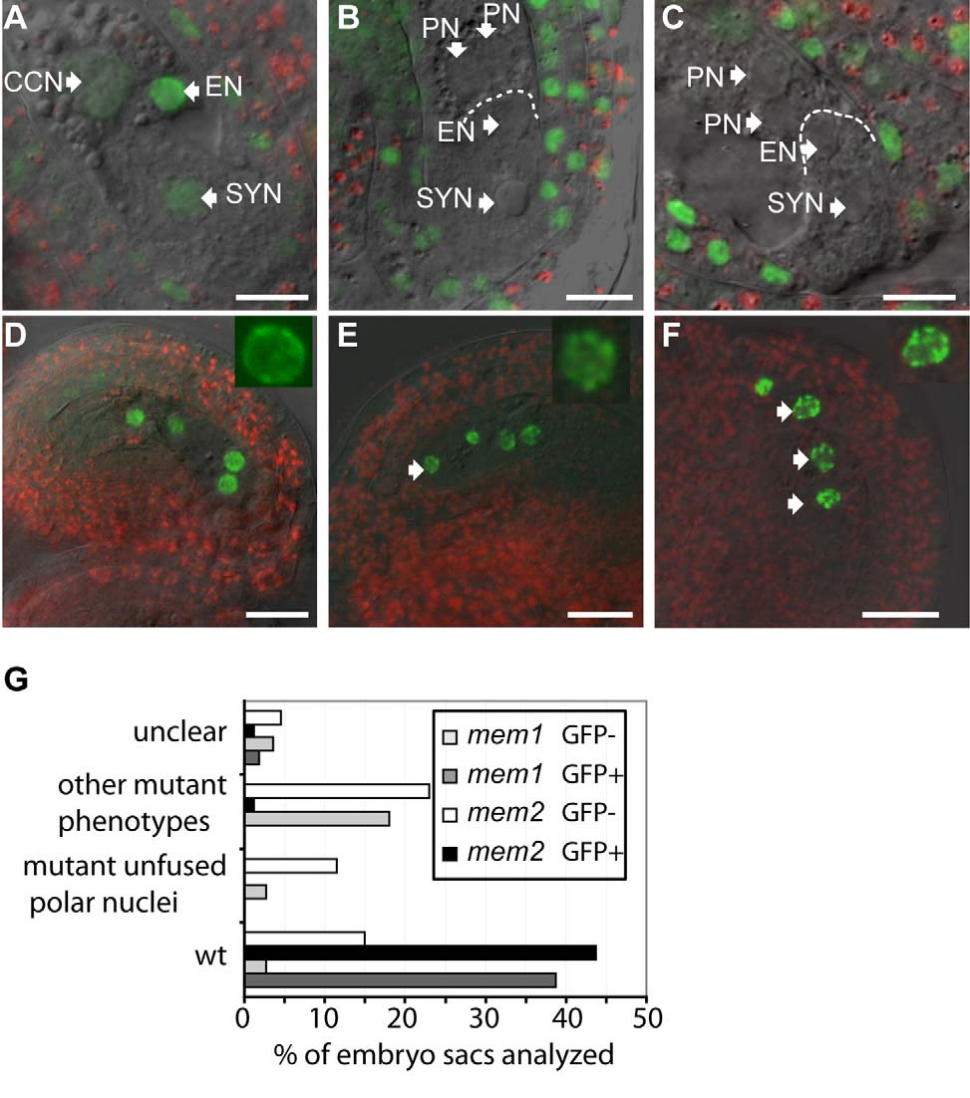
Studies of changes in the epigenetic landscape in *mem-1* and *mem-2* mutant gametophytic nuclei. (A–F) GFP and YFP signals were studied by confocal microscopy. Scale bars are 20 µm. Arrows point to nuclei. (A–C, G) Analysis of *TFL2-GFP* expression under the control of the *TFL2* promoter in *mem* heterozygous plants. Fluorescence of the GFP marker protein was observed in nuclei of central cell (CCN), egg cell (EN), and synergids (SYN), indicating binding of the TFL2-GFP fusion protein to H3K27me3 methylation marks (A). No GFP signals were observed in mutant mature gametophytes with two unfused polar nuclei (PN) in *mem-1* (B, G) and *mem-2* mutants (C, G) and rarely in ovules with other mutant phenotypes (G). (D–F) Analysis of H2B-YFP expression under the control of the *AKV* promoter in plants heterozygous for the *mem-1* (D, E) and *mem-2* allele (F). The *AKV* cell identity marker expressed in developing gametophytes indicates a more condensed heterochromatin structure in some nuclei of mutant gametophytes (arrows point to nuclei; insets: signal distribution in wild-type (D) and mutant nuclei (E, F)). (G) Percentages of mutant phenotypes and presence (+) or absence (−) of GFP signal due to the TFL2-GFP marker observed in a total of *N* = 111 and *N* = 87 mature embryo sacs analyzed from *mem-1/MEM* and *mem-2/MEM* plants, respectively. GFP signal was only occasionally observed in mutant ovules. If embryo sacs could not clearly be classified as mutants or wild-type, they were recorded as “unclear”; if they were clearly mutant but the central cell nuclei/us was/were not visible, they were recorded under “other mutant phenotypes.” The latter class includes 8% of *mem-2/MEM* ovules without GFP signal that likely had unfused polar nuclei, which were, however, not clearly visible. In contrast, GFP signal was observed in 93% and ≥78% of the wild-type ovules in the *mem-1* and *mem-2* mutants, respectively.

Wild-type ovules showed strong signals in the nuclei of synergids and the egg cell, and a weaker signal in the central cell nucleus as recently described ([42]; Figure 7A). We focused on the most abundant mutant class with two unfused polar nuclei, as such embryo sacs were well distinguishable from the wild type while being morphologically closest to the wild type. No GFP signal was detected in *mem-1* and *mem-2* mutant female gametophytes with unfused polar nuclei (*N* = 20 and *N* = 10, respectively) and only rarely in other mutant classes (Figure 7B,C,G). This finding suggests that *MEM* is directly or indirectly involved either in the proper establishment of euchromatic repressive marks in the germline or their interpretation by regulation of *LHP1*.

Changes in the epigenetic setup of a cell might also involve changes in chromatin structure. The H2B-YFP marker under the control of the *AKV* promoter reflects some aspects of chromatin structure during megagametogenesis. In ovules of plants carrying a mutant *mem-1* or *mem-2* allele, we frequently observed a different distribution of H2B-YFP as compared to the wild type, as shown for developing gametophytes with four gametophytic nuclei, which might be derived from two MMC-like cells (Figure 7D–F; Figure S8). Mutant and wild-type gametophytes could be distinguished by the unusual position and size of gametopyhtic nuclei in *mem* gametophytes. In particular, more than five heterochromatic foci indicated by spots of high signal intensity (chromocenters) were often observed in mutant gametophytic nuclei of *mem-1*/*MEM* or *mem-2*/*MEM* plants, changes in chromatin structure we did not observe in gametophytes of wild-type plants expressing this marker (unpublished data), which show a more equal distribution of H2B-YFP not exceeding five chromocenters. These results indicate changes in chromatin structure of gametophytic nuclei and possibly higher ploidy, as more chromocenters were detectable than expected for haploid nuclei. Intriguingly, higher H2B-YFP fluorescence was observed by quantification of the signal intensity in the nuclei of additional FMSs or developing female gametophytes in *mem* mutants as compared to the wild type. In addition, similar differences were observed within one ovule between the FMSs or developing gametophytes in the normal position and the additional FMSs or gametophytes in more micropylar positions (Figure S8), suggesting a higher ploidy of the latter.

In summary, *MEM* plays a role for key steps of plant reproduction, including megasporogenesis, megagametogenesis, and embryogenesis. Importantly, heterozygous plants carrying a mutant *mem-1* or *mem-2* allele were affected in restriction of the germline lineage to one cell per ovule primordium, a phenotype resembling mutants in the small RNA and DNA-methylation pathways, both important for epigenetic regulation [18],[19]. Interestingly, changes in the epigenetic setup of *mem* gametophytic nuclei were observed, providing an explanation for the defects found at later developmental stages when *MEM* expression is not detectable anymore. Nonetheless, a function of the extremely low *MEM* expression levels during early seed development cannot be excluded. The characterization of *MEM* illustrates the usefulness of our MMC transcriptome dataset for the identification of genes and functions important for megasporogenesis and early development of the plant female reproductive lineage.

## Discussion

### Plant and Animal Germ Line Share Regulatory Features

Formation and specification of the MMC is a key step in plant reproduction, marking the developmental switch from the sporophytic (“somatic”) fate to the reproductive or “germline” lineage. To our knowledge, we present the first transcriptome analysis of the MMC and the surrounding nucellus tissue in the sexual model plant *Arabidopsis*. Hierarchical sample clustering revealed that the MMC transcriptome is clearly distinct from that of the surrounding nucellus or the cells of the mature gametophyte. Our data indicate that translational control, ribosome biogenesis, and the expression of DEAD/DEAH-box helicases are major features of MMC specification in plants. This resembles an important feature of the animal germline, where translational regulation is a fundamental and highly conserved mechanism for restricting gene activity (reviewed in [37]). While transcription is active early in gametogenesis in animals, differentiation into sperm cells and oocytes is under translational control as the reproductive cells enter meiosis (reviewed in [50]). Particularly, specific RNA-helicases like Vasa and the eukaryotic translation initiation factor eIF4A, but also the RNA-binding proteins Boule, Bruno, and Pumilio, together with the zinc finger protein Nanos, are determinants of the *Drosophila* germline. They are involved in regulating the maintenance of stem cell fate and the differentiation to the gametes [37]. In flowering plants, similar molecular mechanisms might be required for the transition from undifferentiated meristematic cells of the nucellus to the reproductive fate. Interestingly, *Arabidopsis* homologues or proteins harboring similar functional domains as those described in animals were identified among the genes with specifically enriched expression in the *Arabidopsis* MMC, including the three DEAD/DEAH-box helicases MEM, eIF4A (AT1G72730), and AT3G16840; two PUMILIO (PUM) proteins, PUM7 and PUM23; as well as different RNA-binding and zinc finger proteins (Table S7). These findings suggest that similar regulatory pathways may be involved in germline specification and the development of female gametes in plants and animals. This is in line with the recent finding of shared features of epigenetic regulation through the small RNA pathway in plant and human gametes [22].

Genes involved in different small RNA pathways were found to be expressed during *Arabidopsis* megasporogenesis (Figure S9, Figure S10) and, as previously reported, during male gametogenesis (Figure S9, Figure S10 [51]). Thus, genes involved in small RNA pathways, including members of the *AGO* gene family, appear to play important roles in the regulation of germline development and the maintenance of germline integrity not only in animals but also in plants [52],[53]. However, it remains to be elucidated whether these similarities represent the consequence of convergent evolution or ancestral features. Interestingly, a functional divergence of small RNA pathways has been found between these kingdoms—for example, in their requirements for target recognition [54] and the absence of the PIWI-clade of AGO proteins, whose members are abundantly expressed in the animal germline [55], in plants.

### Mutants Affecting the MEM RNA-Helicase Show Aspects of Apospory

In animals, functional interactions between the RNA-based silencing pathway and the germline-specific Vasa family of RNA-helicases have been described (reviewed in [56]). However, while Vasa and Vasa-like DEAD-box RNA helicases are widely conserved in the animal kingdom, no Vasa proteins have been discovered in plants. Nevertheless, in plants other DEAD/DEAH-box helicases may have similar functions. We analyzed two independent mutant lines with T-DNAs disrupting the *MEM* gene, encoding an RNA helicase with highly specific expression in the MMC. Future studies will be required to elucidate whether *MEM* may be functionally interacting with the small RNA pathway. Interestingly, *mem* mutants affect archespore selection and MMC specification leading to the initiation of two gametophytes in one ovule. These abnormalities resemble recently described *Arabidopsis* mutants involved in the small RNA pathway (*ago9*, *sgs3*, *rdr6*) [18] and maize mutants in the DNA-methylation pathway [19]. Similar to these mutants, an additional enlarged cell in *mem/MEM* ovules may proceed to form a gametophyte without undergoing meiosis, as it occurs in aposporous apomicts [4]. This is consistent with the finding that additional developing FMSs or gametophytes have a higher ploidy than those in the wild-type position. Identification of the molecular players controlling apospory and other components of apomixis is a long-standing goal in plant research, as apomixis leads to the production of clonal offspring, a feature that has important agricultural applications [57]. However, unlike *AGO9*, which has been detected in the somatic cells that form additional MMC-like cells [18], *MEM* shows enriched expression in the MMC, suggesting that non-cell autonomous components regulate germline fate. It has been postulated for a while that the MMC suppresses the development of additional MMCs in a non-cell-autonomous fashion [2], but the molecular components were not known. Nevertheless, though at significantly lower levels, *MEM* expression was detected in the nucellus cells such that cell-autonomous effects in the cells neighboring the MMC cannot be fully excluded.

Although additional embryo sacs are formed, two mature gametophytes within one ovule have neither been observed in *mem* mutants, nor reported for *Arabidopsis* mutants affecting small RNA pathways or maize mutants defective in DNA-methylation [18],[19]. It remains to be elucidated whether the additional gametophytes in *mem* mutants can occasionally give rise to viable offspring. With respect to defects in seed development the above mutants vary, too: while *ago9* and one of the maize mutants (*dmt102::Mu/dmt102::Mu*) are nearly fully fertile, the other maize mutant (D103 RNAi lines against *Dmt103*) shows seed abortion as we observed it for *mem*.

Apart from *MEM*, other DEAD/DEAH-box helicases are enriched in the MMC. These helicases might have distinct or redundant functions. In *Arabidopsis* ATP-dependent RNA-helicases are a large protein family with 78 annotated members, generally involved in unwinding stable RNA (or DNA) duplexes using ATP as a source of energy. RNA-helicases in general are involved in multiple processes of RNA metabolism and play a role in developmental processes including pollen tube guidance, megagametogenesis, and seed development, as already demonstrated for *MAGATAMA* (*MAA*), *Arabidopsis thaliana RNA HELICASE36/SLOW WALKER3* (*RH36/SWA3*), and *FREYA* (*FEY*) [58]–[61]. In addition, *embryo sac development arrest15* (*eda15*) mutant plants, carrying a mutant allele of *AtSUV3*, a gene with homology to *MEM*, develop abnormal numbers of nuclei during gametophyte development [62].

*SUV3* genes are evolutionary highly conserved from purple bacteria to higher eukaryotes including plants and humans [63]. They are involved in unwinding dsDNA, dsRNA, and RNA-DNA heteroduplexes [64]. While SUV3 proteins studied so far are localized predominantly in the mitochondria, the human SUV3 ortholog is partially present in the nucleus and is probably involved in chromatin maintenance, cell-cycle regulation, and the regulation of apoptosis [65]. In the *mem*/*MEM* heterozygous mutants instead of one, two cells with FMS characteristics were often observed. It is also possible that an apoptosis defect in one of the three degenerating megaspores might result in a second surviving FMS-like cell; however, surviving FMS-like cells should have reduced ploidy unlike what we observed.

### Processes during Megasporogenesis Set the Epigenetic Landscape for Later Stages

In summary, the functional analysis of *MEM* revealed structural abnormalities from the onset of megasporogenesis to embryo development, suggesting that *MEM* function is required at several stages of reproductive development. The enriched abundance of *MEM* transcript in the MMC, together with the observed changes in LHP1 binding and chromatin structure in *mem* female gametophytic nuclei, suggests an involvement of *MEM* in establishment of the epigenetic landscape in the female gametophyte. In this way, *MEM* expression during megasporogenesis might be relevant for the regulation of transcriptional control at later stages of reproductive development. The importance of the epigenetic state of the mature gametes for the transition from gametophyte to seed development has recently been demonstrated [42]. Interestingly, the observed changes in chromatin structure in *mem* mutant gametophytic nuclei are in agreement with the functions of the human SUV3 ortholog in chromatin maintenance [65]. Apart from this, the enrichment of genes regulating chromatin structure in the MMC as compared to the mature gametophyte suggests a more general role of epigenetic regulation in the acquisition of germline fate in the female reproductive lineage. Recent studies also provide evidence for an involvement of epigenetic regulation in the differentiation between sexual gametophyte formation and apospory [18],[19]. However, it remains to be seen whether the modifications in epigenetic marks and chromatin structure observed in *mem* mutant gametophytes play a role in this respect. Apart from *MEM*, a number of genes enriched during megasporogenesis as compared to the mature gametophyte play important roles during embryo development, such as *MATERNAL EFFECT EMBRYO ARREST63* (*MEE63*) and several *EMBRYO DEFECTIVE* (*EMB*) genes [62],[66]–[70]. In these cases, gene function might be masked by haplo-sufficiency or redundancy during megasporogenesis and become apparent only during embryonic development. Alternatively, a subset of genes expressed during early stages of reproduction might determine the developmental fate of later stages—for example, by establishing epigenetic marks required for activation or repression of gene expression later in development. However, given the evidence for the importance of translational control during gametophyte development, transcripts present in the MMC might encode proteins whose activities are only required at later stages of reproductive development.

A total of 13 genes were significantly enriched as compared to the tissue atlas including the sporophytic nucellus. This specificity of expression suggests an importance of the gene function for the developing MMC, as demonstrated by the characterization of one of those genes—*MEM*—for MMC specification and gametophyte development. Notably, we found *YUCCA2* and *AT1G29440*, genes involved in auxin synthesis and signaling, enriched in the MMC. An auxin gradient established during megagametogenesis has recently been proposed to be important for cell specification [17]. However, to date no role for auxin has been ascribed for megasporogenesis. As *YUCCA2* expression is modulated by *SPL/NZZ* during lateral organ development [16], it might link homeotic gene function underlying reproductive organ development with gametogenesis.

In conclusion, our study indicates that similar molecular mechanisms are acting upon germline specification and differentiation in animals and in plants. Control of translational regulation is a dominant feature in the transcriptome dataset and RNA processing involving RNA-helicases plays an important role for early stages of female gametophyte development. *MEM*, a gene encoding a helicase with significantly enriched expression in the MMC, plays important roles for restriction of the reproductive fate to only one cell per ovule primordium and for gametophyte development. Thus, this transcriptome analysis of the *Arabidopsis* MMC provides insights into the molecular basis of a key step of plant reproduction. A detailed understanding of the mechanisms underlying megasporogenesis is not only interesting from a fundamental point-of-view, but also the precondition for the manipulation of this pathway towards apomixis, which is of great importance for plant breeding and seed production.

## Materials and Methods

### Plant Material

*Arabidopsis thaliana* (L.) Heynh., accession Landsberg *erecta*, was used for LAM sample preparation, as specimen for in situ hybridizations, and for plant transformation throughout this study. *Arabidopsis thaliana* Col-0 plants were used as wild-type plants in the context of the mutant analysis. Seedlings were grown on MS plates for 7–12 d before transfer to soil (ED73, Universalerde, Germany) and grown in a growth chamber at 16 h light / 8 h darkness at 21°C and 18°C, respectively. Plants were treated with a 10% milk suspension and nematodes against powdery mildew and black flies, respectively. Enhancer trap lines and T-DNA insertions were ordered from the Cold Spring Harbor Trapper Collection (http://genetrap.cshl.edu/) or NASC (http://arabidopsis.info) and grown as described above. p*TFL2*:*TFL2*-*GFP* and p*AKV*:*H2B*-*YFP* marker lines were kindly provided by K. Goto and W.-C. Yang, respectively. The *PUM12*-GUS reporter line was described previously [22]. The *pABCB*19:*ABCB19*-*GFP* (p*PGP19*:*PGP19*-*GFP*) marker line was kindly provided by M. Geisler [25].

### Laser-Assisted Microdissection

To prepare material for LAM, inflorescences were fixed on ice in farmers' fixative (ethanol:acetic acid 3∶1), vacuum infiltrated two times for 15 min, and stored on ice overnight. The fixative was replaced by 70% ethanol and young buds were selected under the dissecting scope. Subsequently, tips of the ovaries were dissected using injection needles, cleared in chloralhydrate:glycerol:water (8∶1∶2; w:v:v), and subjected to microscopic analysis. Buds with ovules harboring MMCs (predominantly before meiosis or at meiosis I) were embedded in Paraplast X-tra in an ASP200 embedding machine (Leica Microsystems, Wetzlar, Germany) as described [22]. Paraplast embedded samples were stored at 4°C until further use.

Thin sections of 6–7 µm were prepared from the samples using a RM2145 Leica microtome and mounted on PET metal frame slides (Molecular Machines and Industries (MMI), Glattbrugg, Switzerland) using methanol. Slides were dried overnight on a heating table at 42°C and subsequently dewaxed two times for 10 min in Xylol (Merck, Darmstadt, Germany). LAM was performed with a SL µCut and a CellCut Plus Instrument (MMI). MMCs and surrounding sporophytic nucellus tissue were subsequently isolated and collected separately on MMI isolation caps. On average, ∼65 MMC sections were collected per day on one isolation cap (estimated to be equivalent to ∼50–55 MMCs). In addition, one or two ovary sections were isolated per slide to control for RNA quality.

### RNA Isolation and Amplification

LAM samples were stored at −80°C until extraction. RNA was isolated using the PicoPure RNA isolation kit (Arcturus Engineering, Mountain View, USA) following the manufacturer's instructions with modifications. For extraction, caps were covered with 10–11 µl of Extraction buffer from the kit, incubated at 42°C for 30 min, and pooled for binding on the column. RNA integrity was tested on a Bioanalyzer (Agilent, Santa Clara, USA), using control sections dissected after collection of MMCs and surrounding nucellar tissue from the same slides. After optimization, RNA integrity was good and reproducible at ∼RIN7. Isolated RNA from ∼560 to 930 pooled sections of MMCs or the surrounding nucellar tissue were subjected to two rounds of linear amplification with the MessageAmpII Kit (Ambion, Foster City, USA), following the manufacturer's instructions. During the second round of amplification, biotin-11-UTP (Ambion) was incorporated in the amplified aRNA for array analysis. Quantity and fragment size distribution of the amplified product was analyzed using a Nanodrop and the Bioanalyzer. Samples with amplification yields between 16 µg and 40 µg were used for samples MMC1 to MMC3; for MMC4, three samples with suboptimal amplification yields between 2.6 and 7.1 µg were pooled. Amplification yields from the sporophytic nucellar samples ranged between 38 µg and 65 µg.

### Array Hybridizations

15 µg labeled aaRNA was fragmented and hybridized onto the *Arabidopsis* ATH1 GeneChip (Affymetrix) for 16 h at 45°C as described in the technical manual. The hybridization, staining, washing, and subsequent array scanning was performed as described previously [22]. Original data-files (.CEL) are deposited on ArrayExpress (http://www.ebi.ac.uk/arrayexpress/) under accession E-MEXP-3137 (megaspore_mothercell, including the four MMC samples) and under E-MEXP-3138 (sporo_nucellus, including the four datasets of the surrounding nucellus).

### RNA In Situ Hybridization

Total RNA was isolated from *Arabidopsis* Col-0 inflorescences using the RNeasy Plant Mini Kit (Qiagen, Hilden, Germany) and treated on column with *DNase*I. The RNA was subsequently reverse transcribed to cDNA using SuperscriptII *Reverse Transcriptase* (Invitrogen, Carlsbad, USA). Fragments for cloning of in situ probes were PCR amplified with *Taq* (Sigma, St Luis, USA); for primer sequences, see Table S10. Fragment cloning and in situ hybridizations were performed as previously described [22] with modifications: In situ hybridizations were performed on 7 or 8 µm thin sections of inflorescences or buds. Pictures were captured on a Leica DMR microscope (Leica Microsystems, Bensheim, Germany), cropped, and processed in Adobe Photoshop Version 8.0.1 (Adobe Systems Inc., San Jose, CA, USA).

### GUS-Reporter Assays

5′ upstream sequences of genes of interest were PCR-amplified from *Arabidopsis thaliana* Col-0 genomic DNA, using primers containing the 5′attB sites (for primer sequences, see Table S10). The PCR products were cloned into pDONR207 (Gateway Cloning, Invitrogen), using site-directed recombination according to the manufacturer's recommendations. The resulting entry clones were recombined with the destination vector pMDC162 (the At2g24500 promoter) or pSS240 (other entry clones) [71],[72], producing the final binary vectors containing the *uidA* reporter gene encoding β-glucuronidase (GUS). Buds were opened and transferred to the GUS reaction buffer for 24–72 h at 37°C (4 mM 5-Bromo-4-chloro-3-indoxyl-beta-D-glucuronic acid cyclohexylammonium salt (Biosynth AG, Staad, Switzerland), 10 mM ETDA, 0.1% Triton X-100, 2 mM potassium ferrocyanide, 2 mM potassium ferricyanide, 100 mM phosphate buffer (pH = 7.2)), dissected, and mounted in clearing solution (1×PBS, 20% lactic acid, 20% glycerol). Wild-type plants were transformed using the floral dip method [73]. At least three independent F1 lines were analyzed per construct. Enhancer trap lines were GUS-stained following the same protocol.

### Characterization of Plant Mutant Lines

Two independent T-DNA insertion lines disrupting *At5g39840* (*mem-1* and *mem-2*; SAIL_182_A07 and SALK_11370, respectively) were analyzed for phenotypes during reproductive development. Developmental arrest during early seed development was counted after opening the silique with injection needles. For histological analysis, ovules and developing seeds were cleared as described above and subjected to microscopic analysis. For analysis of developmental defects during embryo and endosperm development, *mem1/MEM* and wild-type plants were pollinated 2 d after emasculation and siliques were fixed as described after 2, 3, or 4 DAP. Lines *mem*-1 and *mem*-2 were genotyped with primers 5′-GAATTTCATAACCAATCTCGATACAC-3′, 5′-TACTGCAGACCTCACGAAACC-3′, and 5′-GTCGAGTCTGCAGTGTTTTCC-3′, and with primers 5′-CTTTGACGTTGGAGTCCAC-3′ [74], 5′-AATCGAGTGTTTGCAACAACC-3′, and 5′-GCTAACGAGAGTTCAACACCG-3′, respectively. Position of the T-DNA left border was analyzed by sequencing. For analysis of transmission efficiency at least three heterozygous mutant plants per insertion line were crossed as female or male to the wild-type [41]. The progeny from these crosses were genotyped. *pTFL2:TFL2-GFP* and *pAKV:H2B-YFP* marker lines were crossed to heterozygous *mem-1* and *mem-2* mutant plants as female. The F1 and F2 generations of these crosses were used for the analyses. F2 progeny from the cross of the heterozygous mutants with the TFL2 marker line were analyzed for expression of the marker. From the 42 progenies analyzed from the cross with the *mem-1* mutant line, GFP signal was observed in all plants; among the progeny from the cross with *mem-2*, 18 plants out of 23 were clearly GFP positive, one plant was negative, and in the other plants background fluorescence could not be discriminated from signal. This high frequency suggested more than one copy of the *pTFL2:TFL2-GFP* marker in the genome. Thus, a complete or close linkage of the marker to the *mem-1* and *mem-2* alleles is unlikely. In the heterozygous *mem* mutants carrying the *TFL2:TFL2-GFP* marker an unusually high percentage of ovules arrested at early stages of reproductive development were observed (57% and 52% in heterozygous *mem-1* and *mem-2* mutants, respectively). However, phenotypes observed in mature gametophytes resembled the phenotypes of *mem-1/MEM* and *mem-2/MEM* plants.

### Microscopy

For clearing, GUS-staining, and in situ hybridizations, the slides were viewed under a Leica DMR microscope (Leica Microsystems, Bensheim, Germany) and pictures were taken with a digital camera for microscopes (Magnafire model S99802, Optronics, USA). Confocal images were acquired using a Confocal Laser Scanning Microscope (Leica SP2, Leica). GFP or YFP signal and chlorophyll auto-fluorescence were simultaneously acquired with laser excitation 488 nm and emissions of 500–530 nm for GFP and 590–720 nm for chlorophyll. For quantification of fluorescent signals fluorescence intensity of the nuclei expressing H2B-YFP was measured on 3-dimensional reconstructions of confocal series using IMARIS (Bitplane, CH). Contour surfaces were generated for individual nuclei and the intensity sum was used to calculate the relative intensity. The nucleus with the lowest intensity within one ovule was set to 1.

### Data Processing and Analysis

Quality control was performed as previously described [22]. Robust-Multiarray Analysis (RMA, [75]) was performed using the Bioconductor software (Version 2.6, http://www.bioconductor.org) implemented in the statistical software “R” Version 2.10.1 (http://www.r-project.org).

### Array Annotation

Reannotation of an array can significantly alter the interpretation of a microarray dataset [76]. Therefore, we used reannotation information where probes were mapped to predicted gene sequences of the TAIR9 *Arabidopsis* genome release (downloadable at http://brainarray.mbni.med.umich.edu/). The reannotated array targets 21,504 genes, representing around 64% of the *Arabidopsis* genome utilizing 219,079 single probes. From the latter, 1,732 single probes match multiple genes in the genome perfectly and were removed from the mappings for the analysis using the dChip software (see below), which removed 251 probesets from the analysis. The Bioconductor package affxparser [77] was used to generate a new chip description file (.cdf-file) where the multiple mappings had been removed.

### Mappings

Base-level annotations were downloaded from the Bioconductor homepage (Version 2.6), which includes the Gene Ontology (GO) mappings. Protein family (PFAM) and gene family (FAM) information for *Arabidopsis* were downloaded from TAIR9 (http://www.arabidopsis.org).

### Analysis of Gene Enrichment across an *Arabidopsis* Tissue Atlas

We made use of extensive microarray datasets from *Arabidopsis* for comparing the molecular profile of MMCs and gametes in contrast to tissues of the rest of the organism/body. We processed an *Arabidopsis* atlas consisting of mixed tissue and single-cell tissues as described previously [22] and added the following two datasets: (1) laser-microdissected early embryo and endosperm stages (Harada-Goldberg dataset provided by Ryan Christopher Kirkbride: GSE12404 record in GEO (http://www.ncbi.nlm.nih.gov/gds), as used in [76]) and (2) cell-sorted subdomains of the shoot apical meristem [78].

For finding single genes that show enrichment in MMCs or the sporophytic nucellus, log2-transformed dChip expression indexes were imported into R [79]. A linear model was fitted on the data and modified *t* tests, implemented in the limma-package [36], were used to test every contrast of a given cell type against all other tissues/cell types. Genes with an adjusted *p* value smaller than 0.01 in all contrasts (Benjamini-Hochberg adjustment; [38]) were considered significant.

For finding genes that show enrichment in the MMC as compared to cells of the mature female gametophyte [22], the same method as described for the tissue atlas was used, only that RMA was used for processing the data to generate log2-scale expression indexes and genes were identified at a false discovery rate below 0.05 [36]. We firstly applied a pre-filtering step and restricted the analysis to probesets with evidence of expression for at least three out of 13 arrays (four replicates MMC and three replicates each for egg cell, central cell, and the synergids) as analyzed by *At*PANP. After fitting the linear model and identifying differentially expressed genes using the moderated *F*-statistic (at a false discovery rate below 0.05) [36], each contrast of the MMC against egg cell, central cell, and synergids was examined separately: genes significantly upregulated in all three contrasts were selected as “MMC enriched.”

### Heatmaps

Heatmaps were generated using the Bioconductor package gplots [80], using hierarchical agglomerative clustering (complete linkage) and euclidean distance. Heatmaps were based on log2-transformed mean expression values generated by dChip [81], except for the genes differentially expressed in the MMC and the cell types of the mature female gametophyte (Figure 3), where the heatmap was based on log2-scale expression values generated by RMA [75].

### Calculation of Present/Absent *p*-Values

In order to calculate present/absent *p* values we applied a previously described method called *At*PANP [22], which is a modified version of the original PANP method [82]. The method makes use of internal negative control for the ATH1 GeneChip that consists of probes that do not match sequences from the latest *Arabidopsis* genome release anymore. These negative probes were determined via BLAST [83]. For this, probes present on the ATH1 GeneChip but not used in the probeset annotation were queried against the TAIR9 cDNA and BAC databases (downloaded from www.arabidopsis.org), using the standalone BLAST executable function “blastall” Version 2.2.23 (ftp://ftp.ncbi.nih.gov/blast/executables/). Probes that matched either genomic or cDNA sequences with more than two mismatches only were considered reliable measures for background (a total of 1,574 probes). Single negative probes were randomly assembled into sets of 11, thus constituting negative probe sets. We generated a total of 2,000 negative probesets by resampling randomly from the pool of negative probes. Negative probe set signals were then calculated using the RMA algorithm [75], an algorithm that has been shown to be robust for the analysis of data from amplified RNA [84]. An empirical signal background distribution for each individual array was used to determine the probeset signal threshold for a given false-positive rate—as implemented in the pa.calls-function from the Bioconductor package PANP [82]. *p* value calculations on resampled negative probesets were repeated 20 times and averaged in order to get more robust results. A *p* value threshold of 0.02 was considered significant (referred to as “present”) and a transcript considered expressed when called “present” in at least three out of four replicates and marginally expressed when called “present” in at least two out of four replicates. Venn diagrams of present call overlaps were drawn using the software VENNY [85].

### Gene Ontology (GO), Protein Family, and Gene Family Enrichment

For Gene Ontology (GO) analysis we used the Bioconductor package topGO [86]. We used a Fisher's exact test to test for overrepresented GO terms in combination with the function “weight.” We also used a two-sided Fisher's exact test and comparison against the whole array-genome to test for misrepresentation of protein and gene families.

## Supporting Information

Figure S1 Heatmap of expression values for genes in term “embryonic development.” Heatmap of log2 transformed mean expression values for genes significantly enriched in the MMC as compared to the mature gametophyte at a false discovery rate below 5% annotated in “GO:0009790: embryonic development.” This includes a number of genes previously identified as *MATERNAL EFFECT EMBRYO ARREST* (*MEE*) and *EMBRYO DEFECTIVE* (*EMB*) [62],[66]–[70]. Hierarchical clustering of all samples included in the tissue atlas (see Methods) was based on euclidean distance and hierarchical agglomerative clustering. Colors are scaled per row and yellow denotes high expression and blue low expression. Red box: MMC, PDI (PROTEIN DISULFIT ISOMERASE), PRL (PLEIOTROPIC REGULATORY LOCUS). Samples were sorted into groups (from left to the right, for sample description see Materials and Methods): *root tissues*: root_d7, root_d17, root_endodermis, root_stele, root_xylem, root_columella, root_cortex, root_epidermis, root_ground_tissue, root_protophloem, lateral_root_cap, root_artrichobast, root_pericycle, root_companion_cell; *vegetative tissues*: seedling, cotyledon, hypocotyl, young_leaf, early_rosette, leaf, mature leaf, petiole, whole_plant, senescent_leaf, shoot, internode_shoot, cauline_leaf, inflor_shoot; meristem: Meristem_Clavata3 1, Meristem_Fil; *flower and inflorescence*: pedicel, flower_st6, flower_st9, flower_st11, flower_st12, flower_st15, sepal, sepal_st15, petal, petal_st15, stamen, stamen_st15; *reproduction*: pollen_Schmid, pollen_Borges, sperm, carpel, carpel_st15, early_ovules, late_ovules, sporo_nucellus1, megaspore_mothercell1, synergid_cell, egg_cell, central_cell, embryo_proper_globEmb, glob_embryo_apical, glob_embryo_basal, heart_embryo_cot, heart_embryo_root, peripheral_endosperm_globEmb, micropylar_endosperm_globEmb, chalazal_endosperm_globEmb, seed_coat_globEmb, silique_glob_emb, silique_heart_emb, silique_triang_emb, seed_torpedo, seed_walk_stick, seed_early_curl_cot, seed_early_green_cot, seed_green_cot1.
(TIF)

Figure S2 Heatmap of expression signals of DEAD/DEAH box helicases. Heatmap of log2 transformed mean expression values for (putative) DEAD/DEAH box helicases significantly enriched in the MMC as compared to the mature gametophyte at a false discovery rate below 5%. Hierarchical clustering of all samples included in the tissue atlas (see Materials and Methods, see Figure S1) was based on euclidean distance and hierarchical agglomerative clustering. Colors are scaled per row and yellow denotes high expression and blue low expression (red box: MMC).
(TIF)

Figure S3 Heatmap of expression signals of genes with preferential expression in the sporophytic nucellus. Heatmap of log2 transformed mean expression values for 49 genes significantly enriched in the sporo_nucellus samples as compared to the tissue atlas including the MMC (*p* value <0.01 after Benjamini-Hochberg adjustment, red box: sporo_nucellus). Hierarchical clustering of genes/samples was based on euclidean distance and hierarchical agglomerative clustering. Colors are scaled per row and yellow denotes high expression and blue low expression.
(TIF)

Figure S4 Heatmap of expression signals of genes with preferential expression in MMCs. Heatmap of mean expression values showing 82 genes significantly enriched in the MMC samples as compared to the tissue atlas (*p*-value <0.01 after Benjamini-Hochberg adjustment, red box: MMC). Hierarchical clustering of genes/samples was based on euclidean distance and hierarchical agglomerative clustering. Colors are scaled per row and yellow denotes high expression and blue low expression.
(TIF)

Figure S5 Analysis of *MEM* expression by in situ hybridization. In situ hybridization showing expression of *MEM* in the archespore (arrow points to archespore) (A), the MMC (likely before meiosis, arrow points to MMC) (B), and the endosperm (D, E) of young seeds (30 HAP) in wild-type plants using an antisense probe targeting *MEM*, but not in controls using a sense probe (C, F). A faint signal was also detected in the embryo and the sporophytic seed tissue (D) using the antisense but not the sense probe (F). (A–F) Scale bars 20 µm; embryo (emb); endosperm (end).
(TIF)

Figure S6 Phenotypic analysis of *mem-1* and *mem-2* mutant plants during megagametogenesis. Developmental phenotypes in heterozygous *mem-1* and *mem-2* mutants analyzed by clearing ((A–H) scale bars 40 µm, arrows point towards gametophytic nuclei, ii (inner integument), egg (egg cell), syn (synergids), cc (central cell)) and confocal microscopy ((I–L) scale bars 20 µm). (A–D) Structural abnormalities during early gametogenesis in *mem-1* mutant gametophytes. The unusual shapes of 2-nucleate (A) and 4-nucleate (B) gametophytes were frequently and rarely observed, respectively. In some cases gametophytes develop in unusual positions in the ovule (D,C). (E–G) Mature *mem-2* mutant gametophytes. Gametophytes without discernible gametophytic cells (E), gametophytes with unfused polar nuclei (F), and slim shaped gametophytes (G) were frequently observed. (H) Mature *mem-1* mutant gametophyte with unfused polar nuclei and abnormal positioning of gametophytic nuclei. (I–L) Analysis of H2B-YFP expression under the control of the *ANTIKEVORKIAN* (*AKV*) promoter in *mem-1* (I–K) or the *mem-2* mutant gametophytes (L). The *AKV* cell identity marker indicates three gametophytic nuclei in one ovule belonging to two developing gametophytes (I–K) and development of gametophytes with abnormal positioning of gametopyhtic nuclei.
(TIF)

Figure S7 Phenotypic analysis of *mem-1/MEM* mutant seeds during early stages of embryogenesis. Developmental stages observed during embryogenesis in wild-type and *mem-1/MEM* plants 2 DAP (A) and 3 DAP (B). (C–F) Scale bars 40 µm; embryo (emb); endosperm (end); * indicates degenerated zygote or embryo. (A–E) Presumptive *mem-1* mutant seeds were delayed in embryo and endosperm development as compared to wild-type seeds, with a high percentage of seeds arresting and collapsing around the first division of the embryo (B, F). At 2 DAP, the majority of wild-type seeds contain a 2- or 4-cell embryo proper (A, E), while in siliques of *mem-1/MEM* plants a higher percentage of undivided zygotes (C) and one-cell embryo proper (D) were observed. (B, F) At 3 DAP, early arrested seeds in *mem-1/MEM* mutants started to collapse and degenerate. Only about 10% of developmentally delayed embryos had developed a 2- or 4- cell embryo proper at 3 DAP.
(TIF)

Figure S8 Quantification of fluorescence intensity in ovules with single and double gametophytes to estimate ploidy levels. Relative YFP fluorescence intensity as quantified in gametophytic nuclei of *mem-1* and *mem-2* heterozygous mutant plants harboring either one or two developing gametophytes expressing the H2B-YFP marker under the control of the *AKV* promoter. Wild-type (WT) picture: wild-type ovule at FMS stage in *mem-1/MEM*; other pictures from *mem-2/MEM* mutants; scale bars 20 µm. In ovules harboring two distinct gametophytes at early stages of megagametogenesis, the relative signal intensity from the first gametophyte (developing at the normal position) and the second, additional gametophyte (usually developing in a more micropylar region) differ: a similar intensity as in WT was observed in the first gametophyte, while a higher intensity was observed in the second gametophyte (labeled by *). While in WT the ploidy level of gametophytic nuclei is haploid, the higher signal intensity in additional gametophytes indicates a higher ploidy level, suggesting that these additional gametophytes developed from a somatic cell without meiotic reduction. Importantly, this effect was already observed in ovules harboring two FMS-like cells, but also ovules with four gametophytic nuclei (in *mem* mutants likely belonging to two embryo sacs), making it unlikely that the increase in fluorescence level results from alterations during the first mitotic division of gametophytic nuclei.
(TIF)

Figure S9 Expression of genes involved in DNA methylation and small RNA pathways in selected samples. Heatmap of log2 transformed expression values for 69 genes involved in DNA methylation and different small RNA pathways [51],[53],[87]–[91] of selected cell and tissue types from the female and male germ line lineages and embryogenesis as represented in the tissue atlas, plus additional samples of male gametogenesis [92]. Expression of genes relevant in different small RNA pathways (reviewed in [53]) are active in the MMC, including different members of the *AGO* gene family. The datasets from megasporogenesis (MMC and sporo_nucellus) cluster closer to the datasets from early stages of microgametogenesis (uninucleate microspore and bicellular pollen) and group separately from the mature female gametophyte, gametes, and globular embryo proper. In addition, pollen and sperm group separately from the other samples analyzed. Hierarchical clustering of genes/samples was based on euclidean distance and hierarchical agglomerative clustering. Colors are scaled per row and yellow denotes high expression and blue low expression.
(TIF)

Figure S10 Expression of genes involved in DNA methylation and small RNA pathways across the tissue atlas. Heatmap of log2 transformed expression values for 69 genes involved in DNA methylation and different small RNA pathways (see Figure S9) of all samples composing the tissue atlas of plus additional samples of male gametogenesis [92]. The datasets of mature pollen cluster closer to a variety of sporophytic tissue samples and to endosperm and seed coat, and group separately from different reproductive tissues and cell types, including flowers, siliques, seeds, embryo, ovules, samples from megasporogenesis, cells composing the female gametophyte, and sperm, but also meristems, carpels, and inflor_shoot. Within this subgroup, sperm is distinct from the other samples. Hierarchical clustering of genes/samples was based on euclidean distance and hierarchical agglomerative clustering. Colors are scaled per row and yellow denotes high expression and blue low expression.
(TIF)

Table S1 *At*PANP expression calls. Datasheet with *At*PANP present and absent calls and *p* values in the MMC (megaspore_mothercell), the surrounding nucellus (sporo_nucellus), and the following samples included in the tissue atlas [22]: egg_cell, central_cell, pollen_Borges, sperm, synergid_cell, early_ovules, late_ovules.
(ZIP)

Table S2 Evidence of expression of genes selected for data validation (Figure 2). *At*PANP present and absent calls and mean expression values as calculated by dChip [81].
(DOC)

Table S3 Gene ontology analysis. Analysis of molecular functions upregulated based on the 796 genes enriched in the MMC transcriptome as compared to the transcriptomes of egg cell, central cell, and synergids (*p* value <0.01).
(DOC)

Table S4 Enrichment of PFAM-domains analyzed for genes with preferential MMC expression. Enrichment of protein families was tested by a two-sided Fischer exact test. *P* values <0.01 for genes with significantly higher expression in the MMC in each contrast as compared to the cells of the mature female gametophyte (egg cell, central cell, synergids) were considered significant.
(DOC)

Table S5 Analysis of enriched genes in the sporo_nucellus as compared to the tissue atlas. Mean expression levels calculated by dCHIP [81] for all genes identified to be significantly higher expressed in the sporo_nucellus as compared to the tissue atlas not including the MMC available as additional datasheet. Expression levels in all samples composing the tissue atlas (including the MMC) are given.
(XLS)

Table S6 Gene ontology analysis. Analysis of molecular functions and biological process upregulated based on the 134 genes enriched in the sporo_nucellus transcriptome as compared to the transcriptomes of the tissue atlas not including the MMC (*p* value <0.01).
(DOC)

Table S7 Analysis of enriched gene expression in the MMC as compared to the tissue atlas. Mean expression levels calculated by dChip [81] for all genes identified to be significantly higher expressed in the MMC as compared to the tissue atlas not including the sporo_nucellus available as additional datasheet. Expression levels in all samples composing the tissue atlas (including sporo_nucellus) are given.
(XLS)

Table S8 Gene ontology analysis of MMC enriched genes. Gene ontology analysis to identify biological processes and molecular functions upregulated in 82 genes enriched in the MMC transcriptome as compared to the tissue atlas (sporo_nucellus sample excluded, adjusted *p* value in each contrast <0.01).
(DOC)

Table S9 *At*PANP present and absent calls and *p* values for *MEM. At*PANP present and absent calls and *p* values for *MEM* for all samples of the tissue atlas not included in Table S1 and additional samples from microgametogenesis [92] available as additional datasheet.
(XLS)

Table S10 Primers for cloning. List of primers used for cloning of in situ probes and promoter-GUS expression constructs.
(DOC)

## Abbreviations

emb: embryo
cc: central cell
DAP: days after pollination
FMS: functional megaspore
GUS: ß-glucuronidase
ii: inner integuments
LAM: laser-assisted microdissection
MMC: megaspore mother cell
*MEM*: *MNEME*
nuc: nucellus
n: nucleus
pn: polar nuclei
sporo_nucellus: sporophytic nucellus
