# Supplementary figures and images for: Transcriptome Analysis of the Arabidopsis Megaspore Mother Cell Uncovers the Importance of RNA Helicases for Plant Germline Development
Source: PLoS Biol. 2011 Sep 20;9(9):e1001155. doi: 10.1371/journal.pbio.1001155 (PMC3176755; doi:10.1371/journal.pbio.1001155)

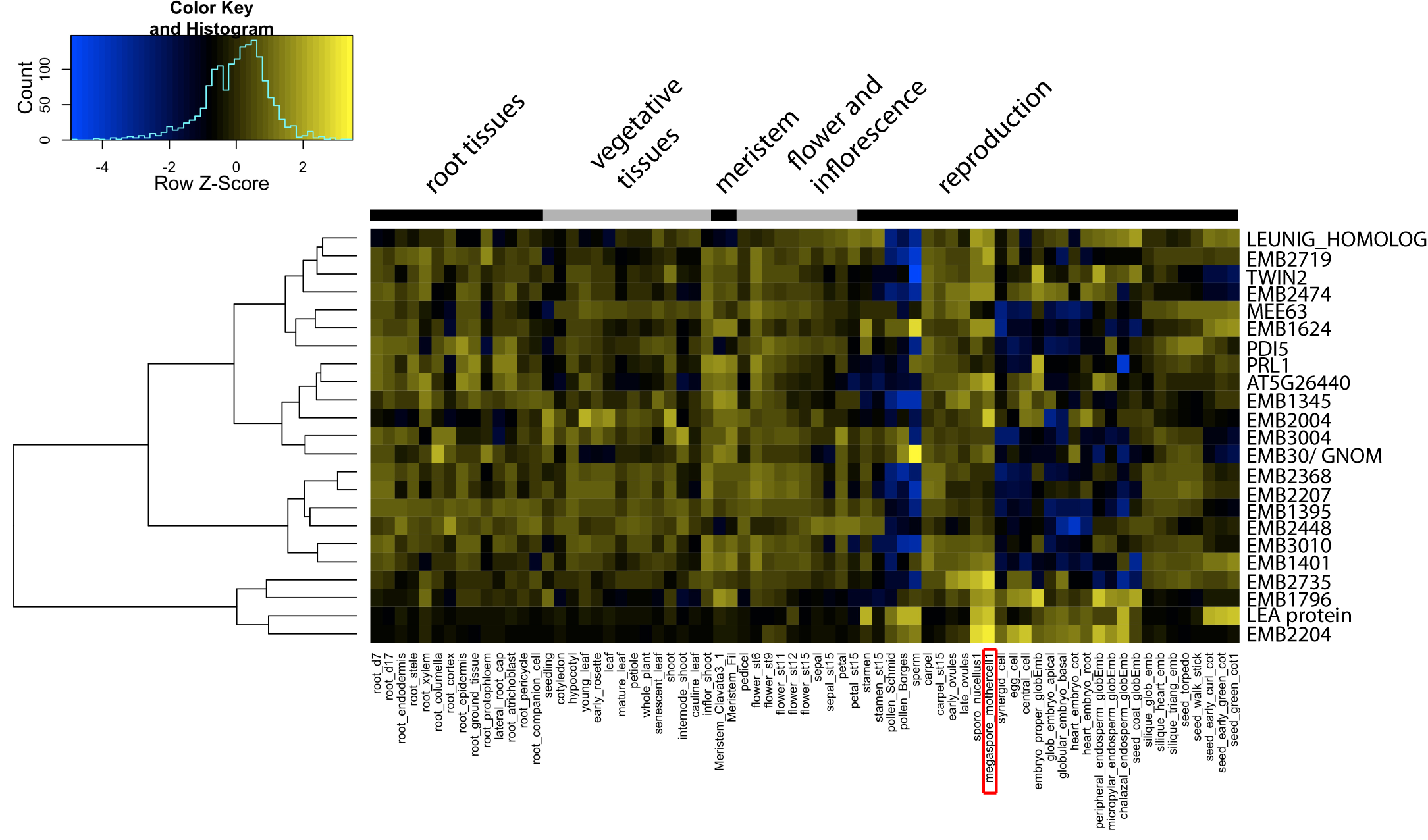

Supplement: Figure S1 — Heatmap of expression values for genes in term “embryonic development.” Heatmap of log2 transformed mean expression values for genes significantly enriched in the MMC as compared to the mature gametophyte at a false discovery rate below 5% annotated in “GO:0009790: embryonic development.” This includes a number of genes previously identified as MATERNAL EFFECT EMBRYO ARREST (MEE) and EMBRYO DEFECTIVE (EMB) [62],[66]–[70]. Hierarchical clustering of all samples included in the tissue atlas (see Methods) was based on euclidean distance and hierarchical agglomerative clustering. Colors are scaled per row and yellow denotes high expression and blue low expression. Red box: MMC, PDI (PROTEIN DISULFIT ISOMERASE), PRL (PLEIOTROPIC REGULATORY LOCUS). Samples were sorted into groups (from left to the right, for sample description see Materials and Methods): root tissues: root_d7, root_d17, root_endodermis, root_stele, root_xylem, root_columella, root_cortex, root_epidermis, root_ground_tissue, root_protophloem, lateral_root_cap, root_artrichobast, root_pericycle, root_companion_cell; vegetative tissues: seedling, cotyledon, hypocotyl, young_leaf, early_rosette, leaf, mature leaf, petiole, whole_plant, senescent_leaf, shoot, internode_shoot, cauline_leaf, inflor_shoot; meristem: Meristem_Clavata3 1, Meristem_Fil; flower and inflorescence: pedicel, flower_st6, flower_st9, flower_st11, flower_st12, flower_st15, sepal, sepal_st15, petal, petal_st15, stamen, stamen_st15; reproduction: pollen_Schmid, pollen_Borges, sperm, carpel, carpel_st15, early_ovules, late_ovules, sporo_nucellus1, megaspore_mothercell1, synergid_cell, egg_cell, central_cell, embryo_proper_globEmb, glob_embryo_apical, glob_embryo_basal, heart_embryo_cot, heart_embryo_root, peripheral_endosperm_globEmb, micropylar_endosperm_globEmb, chalazal_endosperm_globEmb, seed_coat_globEmb, silique_glob_emb, silique_heart_emb, silique_triang_emb, seed_torpedo, seed_walk_stick, seed_early_curl_cot, seed_early_green_cot, see [file pbio.1001155.s001.tif]

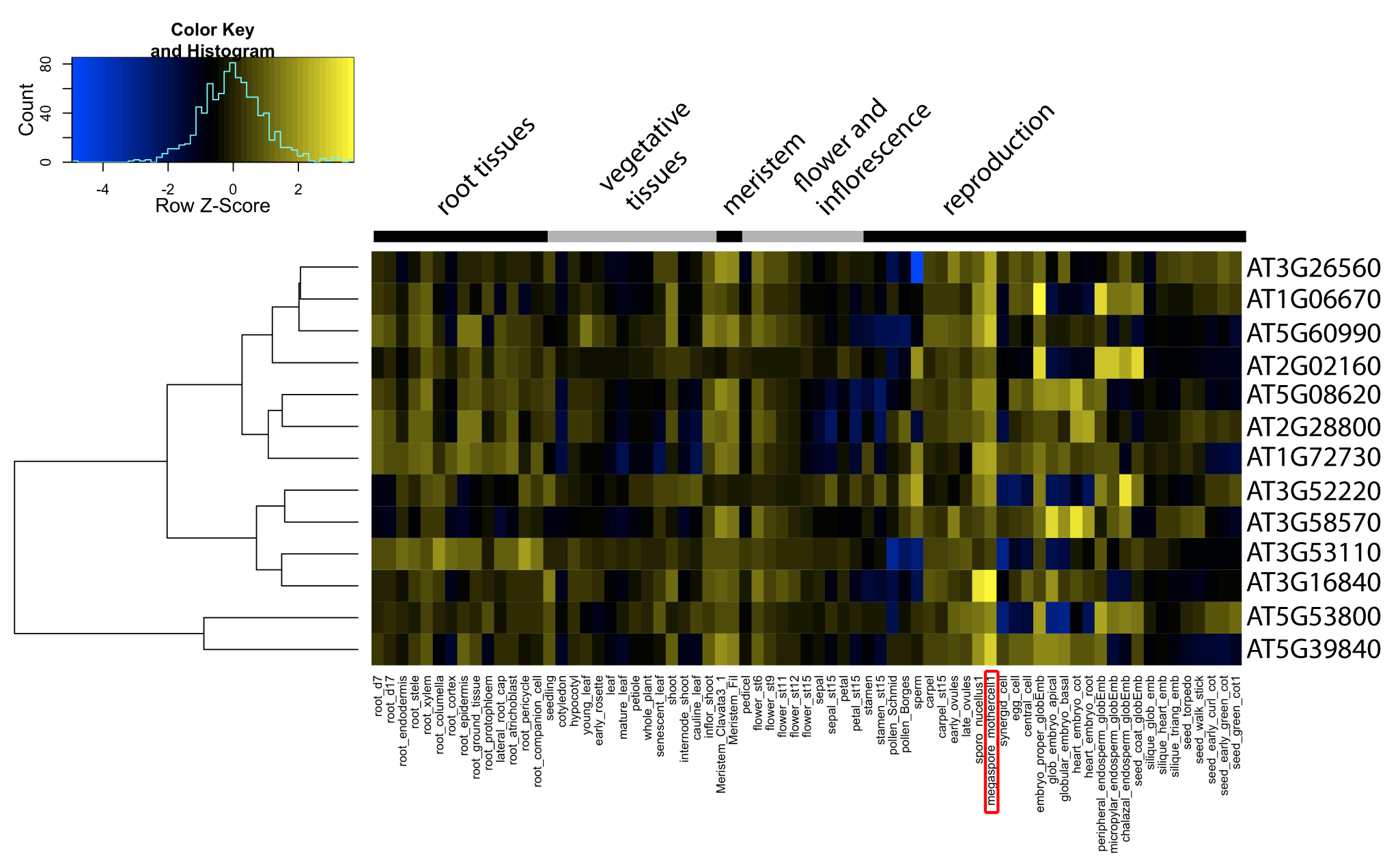

Supplement: Figure S2 — Heatmap of expression signals of DEAD/DEAH box helicases. Heatmap of log2 transformed mean expression values for (putative) DEAD/DEAH box helicases significantly enriched in the MMC as compared to the mature gametophyte at a false discovery rate below 5%. Hierarchical clustering of all samples included in the tissue atlas (see Materials and Methods, see Figure S1) was based on euclidean distance and hierarchical agglomerative clustering. Colors are scaled per row and yellow denotes high expression and blue low expression (red box: MMC). (TIF) [file pbio.1001155.s002.tif]

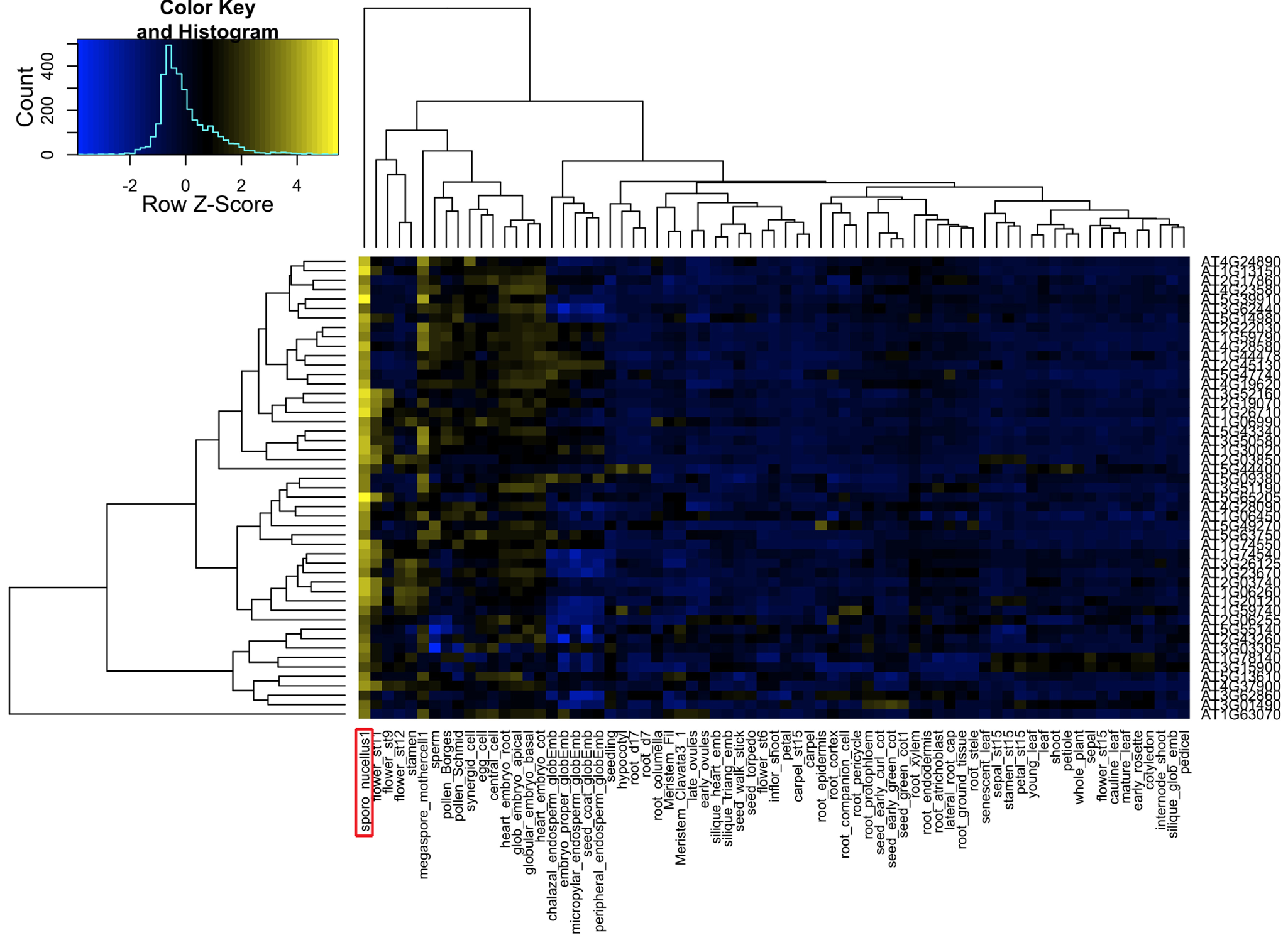

Supplement: Figure S3 — Heatmap of expression signals of genes with preferential expression in the sporophytic nucellus. Heatmap of log2 transformed mean expression values for 49 genes significantly enriched in the sporo_nucellus samples as compared to the tissue atlas including the MMC (p value <0.01 after Benjamini-Hochberg adjustment, red box: sporo_nucellus). Hierarchical clustering of genes/samples was based on euclidean distance and hierarchical agglomerative clustering. Colors are scaled per row and yellow denotes high expression and blue low expression. (TIF) [file pbio.1001155.s003.tif]

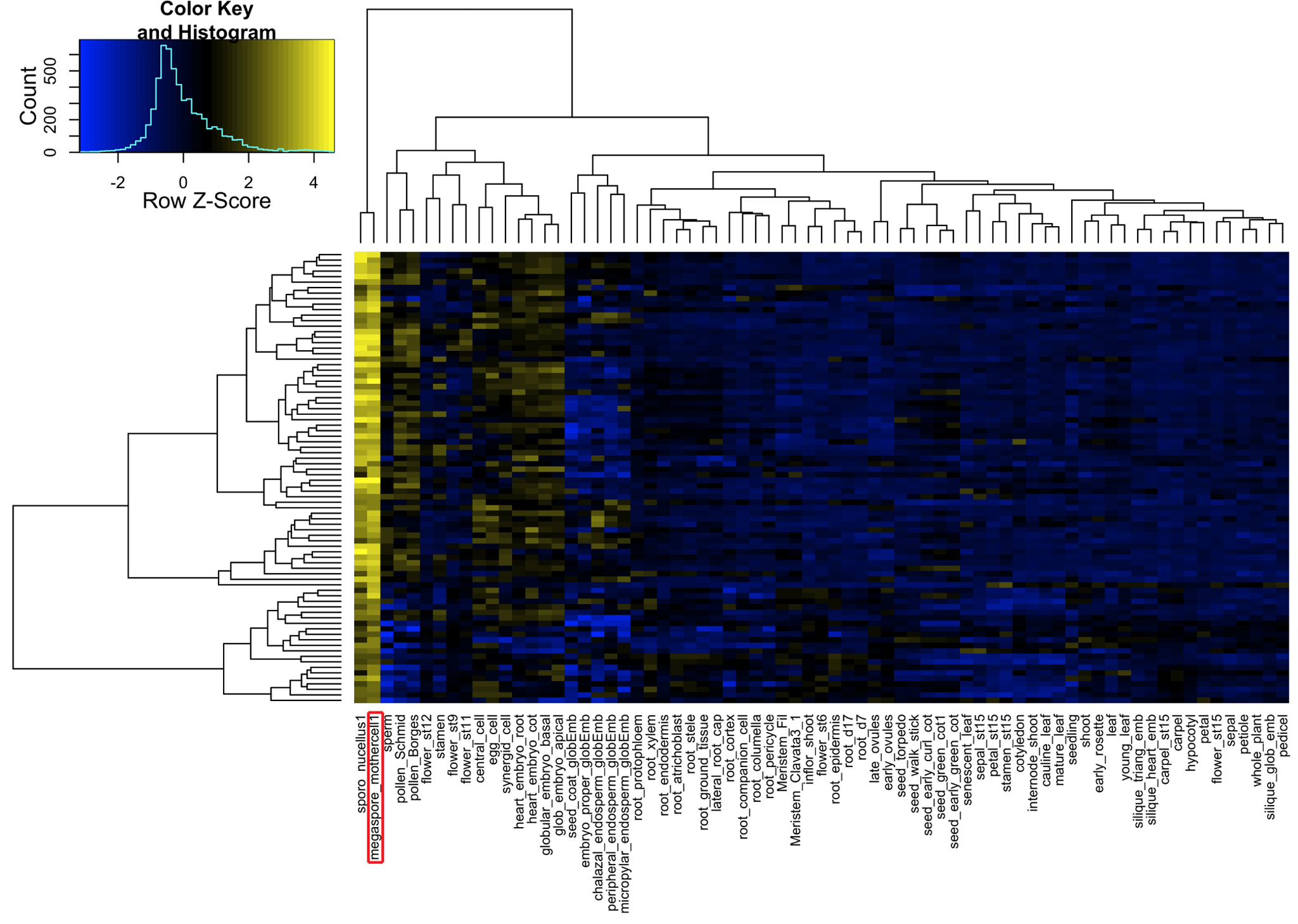

Supplement: Figure S4 — Heatmap of expression signals of genes with preferential expression in MMCs. Heatmap of mean expression values showing 82 genes significantly enriched in the MMC samples as compared to the tissue atlas (p-value <0.01 after Benjamini-Hochberg adjustment, red box: MMC). Hierarchical clustering of genes/samples was based on euclidean distance and hierarchical agglomerative clustering. Colors are scaled per row and yellow denotes high expression and blue low expression. (TIF) [file pbio.1001155.s004.tif]

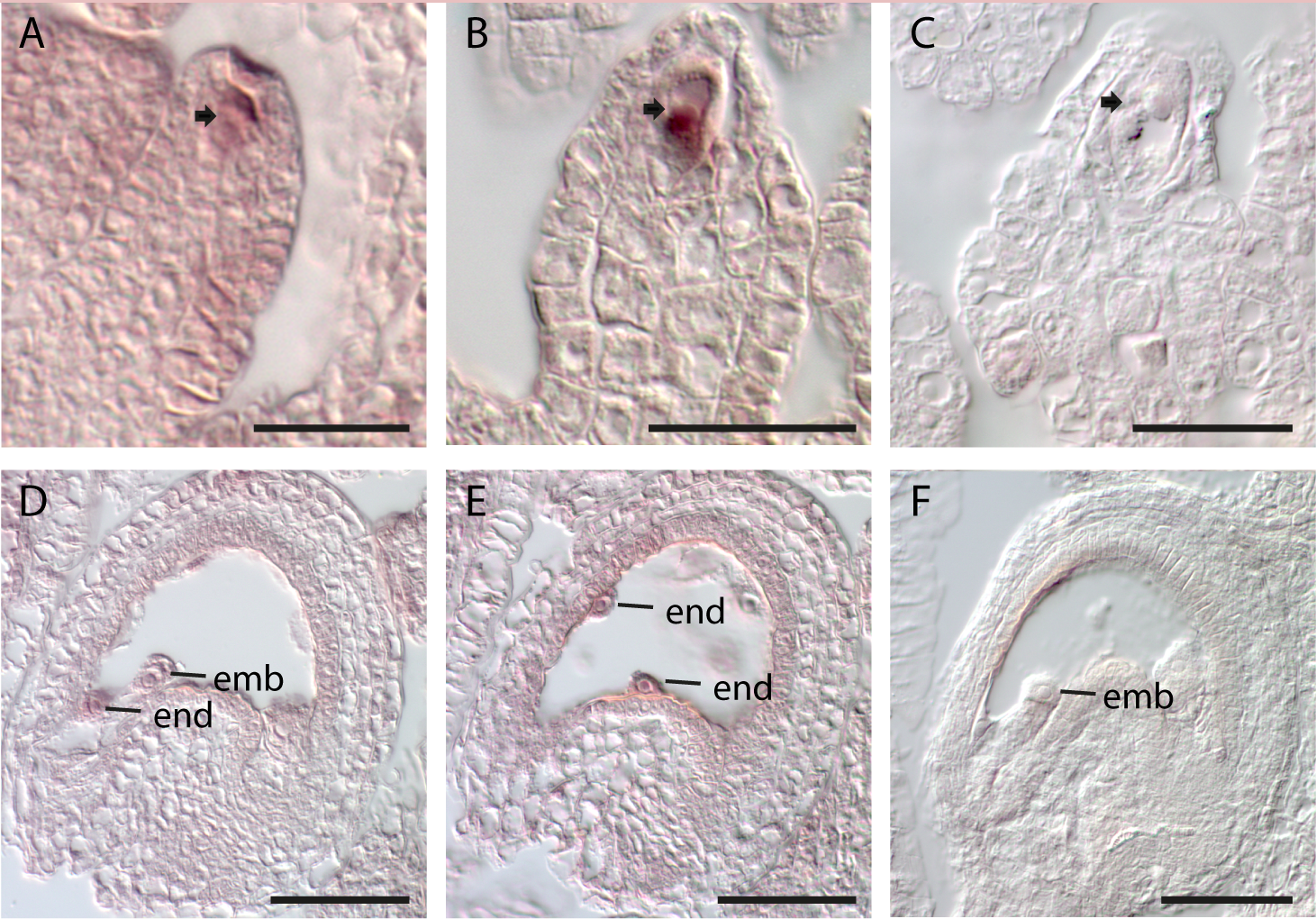

Supplement: Figure S5 — Analysis of MEM expression by in situ hybridization. In situ hybridization showing expression of MEM in the archespore (arrow points to archespore) (A), the MMC (likely before meiosis, arrow points to MMC) (B), and the endosperm (D, E) of young seeds (30 HAP) in wild-type plants using an antisense probe targeting MEM, but not in controls using a sense probe (C, F). A faint signal was also detected in the embryo and the sporophytic seed tissue (D) using the antisense but not the sense probe (F). (A–F) Scale bars 20 µm; embryo (emb); endosperm (end). (TIF) [file pbio.1001155.s005.tif]

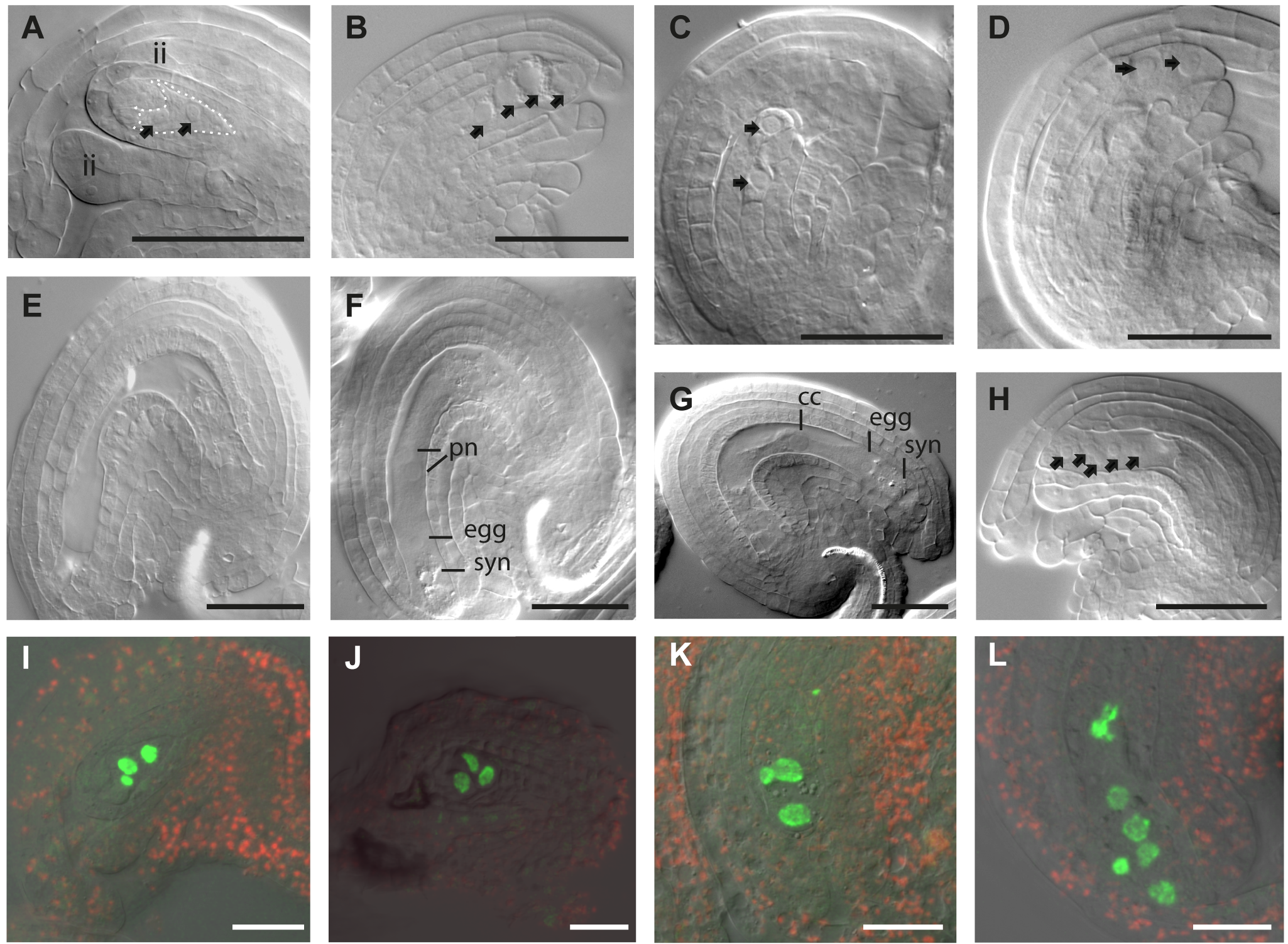

Supplement: Figure S6 — Phenotypic analysis of mem-1 and mem-2 mutant plants during megagametogenesis. Developmental phenotypes in heterozygous mem-1 and mem-2 mutants analyzed by clearing ((A–H) scale bars 40 µm, arrows point towards gametophytic nuclei, ii (inner integument), egg (egg cell), syn (synergids), cc (central cell)) and confocal microscopy ((I–L) scale bars 20 µm). (A–D) Structural abnormalities during early gametogenesis in mem-1 mutant gametophytes. The unusual shapes of 2-nucleate (A) and 4-nucleate (B) gametophytes were frequently and rarely observed, respectively. In some cases gametophytes develop in unusual positions in the ovule (D,C). (E–G) Mature mem-2 mutant gametophytes. Gametophytes without discernible gametophytic cells (E), gametophytes with unfused polar nuclei (F), and slim shaped gametophytes (G) were frequently observed. (H) Mature mem-1 mutant gametophyte with unfused polar nuclei and abnormal positioning of gametophytic nuclei. (I–L) Analysis of H2B-YFP expression under the control of the ANTIKEVORKIAN (AKV) promoter in mem-1 (I–K) or the mem-2 mutant gametophytes (L). The AKV cell identity marker indicates three gametophytic nuclei in one ovule belonging to two developing gametophytes (I–K) and development of gametophytes with abnormal positioning of gametopyhtic nuclei. (TIF) [file pbio.1001155.s006.tif]

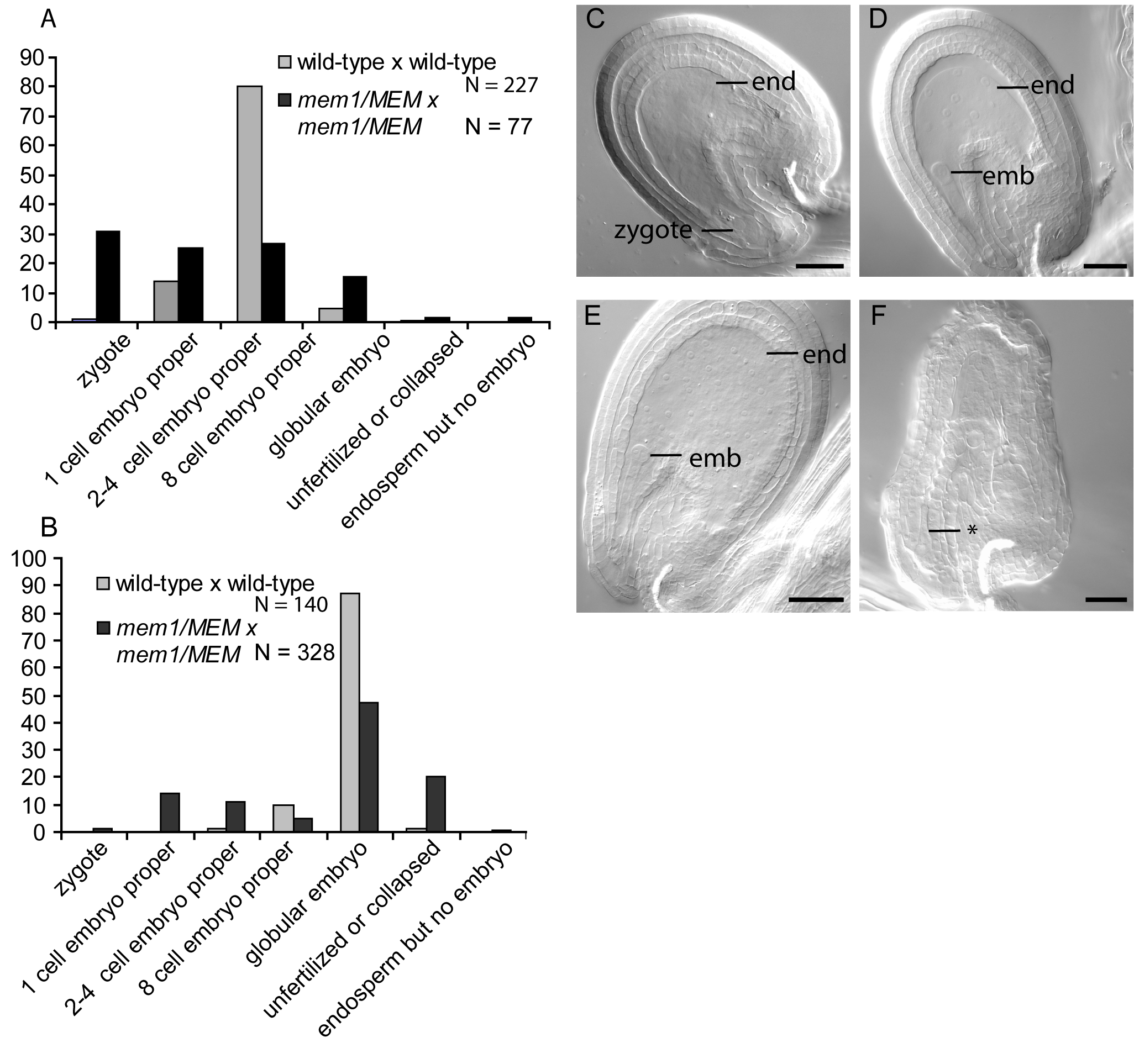

Supplement: Figure S7 — Phenotypic analysis of mem-1/MEM mutant seeds during early stages of embryogenesis. Developmental stages observed during embryogenesis in wild-type and mem-1/MEM plants 2 DAP (A) and 3 DAP (B). (C–F) Scale bars 40 µm; embryo (emb); endosperm (end); * indicates degenerated zygote or embryo. (A–E) Presumptive mem-1 mutant seeds were delayed in embryo and endosperm development as compared to wild-type seeds, with a high percentage of seeds arresting and collapsing around the first division of the embryo (B, F). At 2 DAP, the majority of wild-type seeds contain a 2- or 4-cell embryo proper (A, E), while in siliques of mem-1/MEM plants a higher percentage of undivided zygotes (C) and one-cell embryo proper (D) were observed. (B, F) At 3 DAP, early arrested seeds in mem-1/MEM mutants started to collapse and degenerate. Only about 10% of developmentally delayed embryos had developed a 2- or 4- cell embryo proper at 3 DAP. (TIF) [file pbio.1001155.s007.tif]

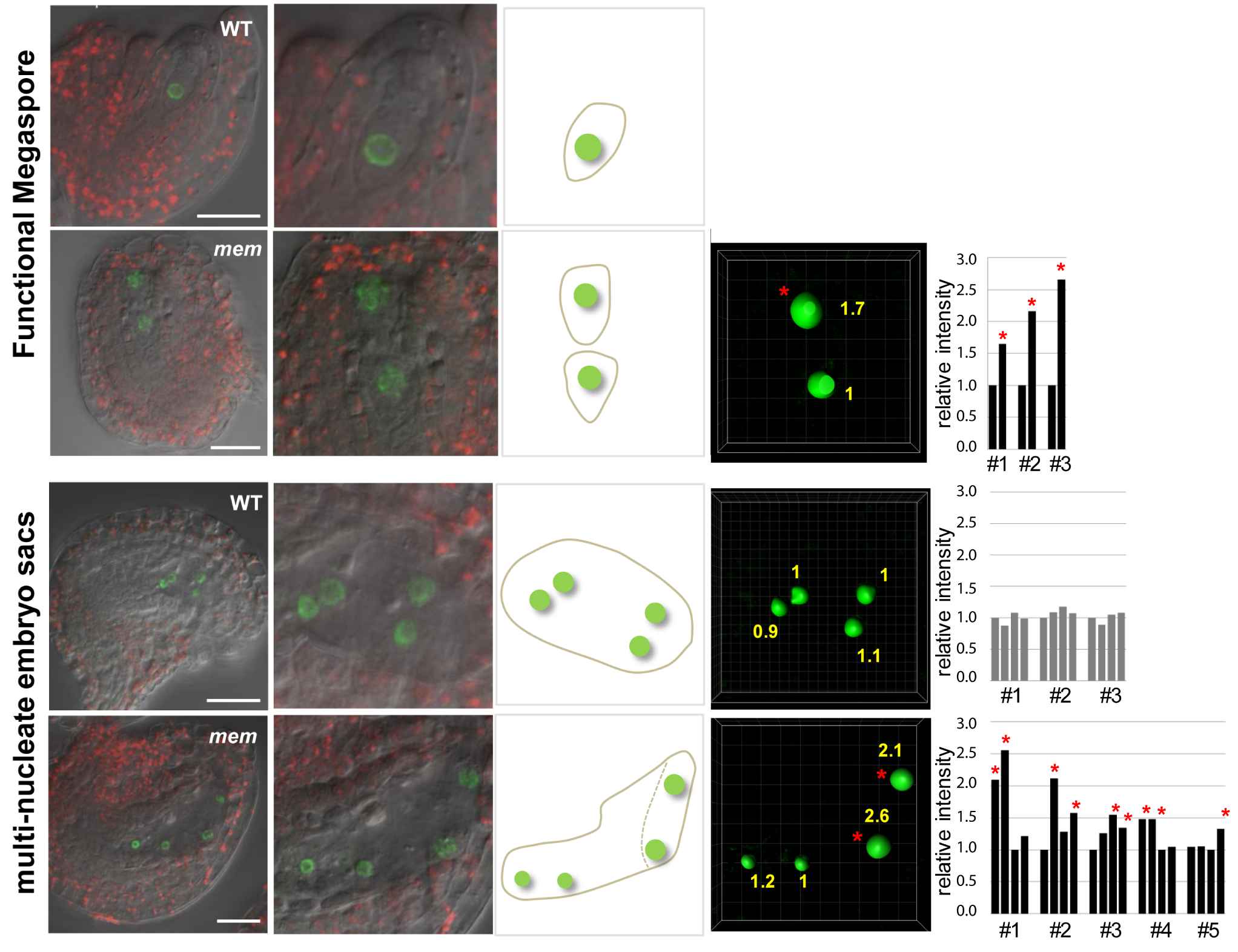

Supplement: Figure S8 — Quantification of fluorescence intensity in ovules with single and double gametophytes to estimate ploidy levels. Relative YFP fluorescence intensity as quantified in gametophytic nuclei of mem-1 and mem-2 heterozygous mutant plants harboring either one or two developing gametophytes expressing the H2B-YFP marker under the control of the AKV promoter. Wild-type (WT) picture: wild-type ovule at FMS stage in mem-1/MEM; other pictures from mem-2/MEM mutants; scale bars 20 µm. In ovules harboring two distinct gametophytes at early stages of megagametogenesis, the relative signal intensity from the first gametophyte (developing at the normal position) and the second, additional gametophyte (usually developing in a more micropylar region) differ: a similar intensity as in WT was observed in the first gametophyte, while a higher intensity was observed in the second gametophyte (labeled by *). While in WT the ploidy level of gametophytic nuclei is haploid, the higher signal intensity in additional gametophytes indicates a higher ploidy level, suggesting that these additional gametophytes developed from a somatic cell without meiotic reduction. Importantly, this effect was already observed in ovules harboring two FMS-like cells, but also ovules with four gametophytic nuclei (in mem mutants likely belonging to two embryo sacs), making it unlikely that the increase in fluorescence level results from alterations during the first mitotic division of gametophytic nuclei. (TIF) [file pbio.1001155.s008.tif]

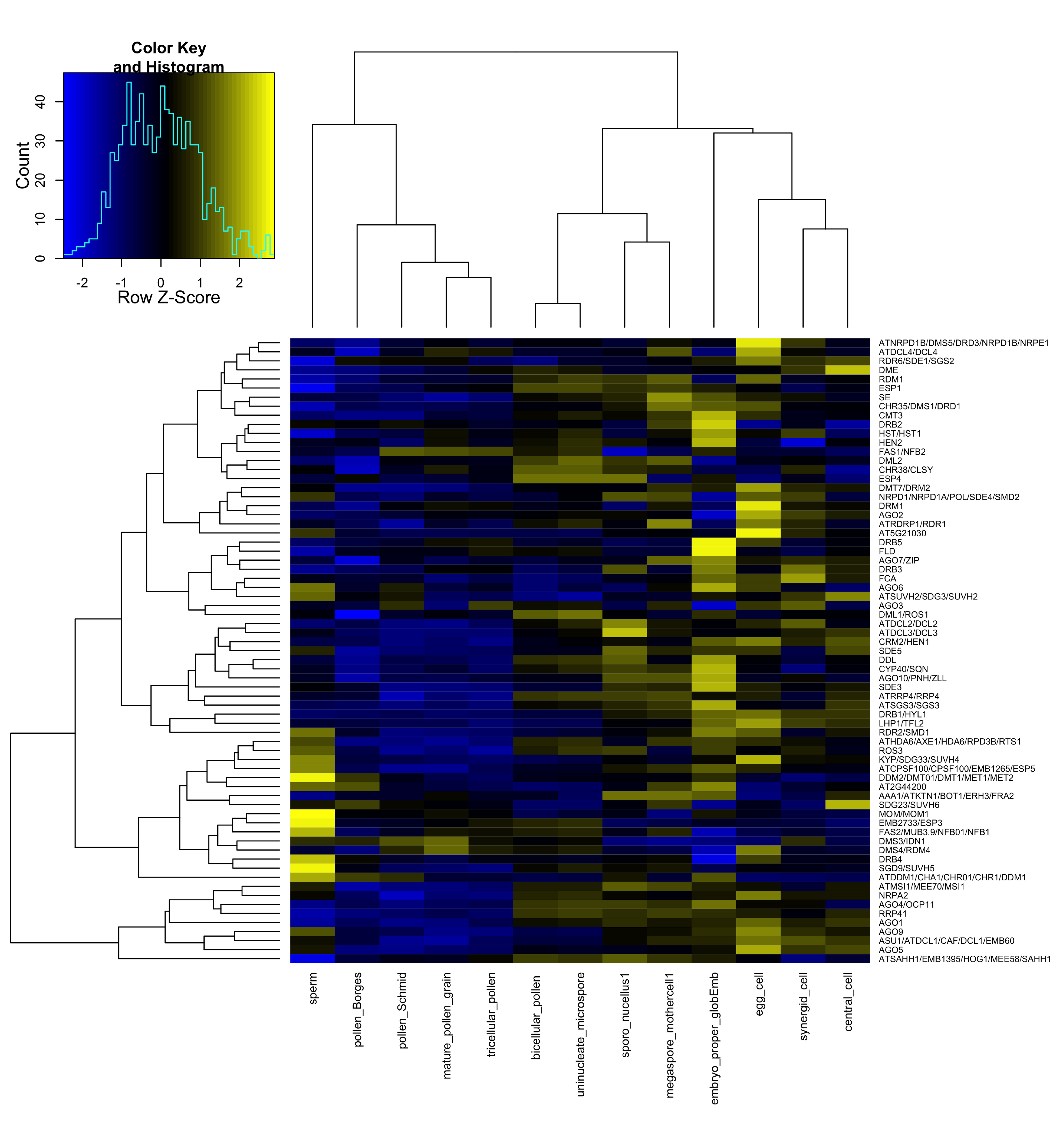

Supplement: Figure S9 — Expression of genes involved in DNA methylation and small RNA pathways in selected samples. Heatmap of log2 transformed expression values for 69 genes involved in DNA methylation and different small RNA pathways [51],[53],[87]–[91] of selected cell and tissue types from the female and male germ line lineages and embryogenesis as represented in the tissue atlas, plus additional samples of male gametogenesis [92]. Expression of genes relevant in different small RNA pathways (reviewed in [53]) are active in the MMC, including different members of the AGO gene family. The datasets from megasporogenesis (MMC and sporo_nucellus) cluster closer to the datasets from early stages of microgametogenesis (uninucleate microspore and bicellular pollen) and group separately from the mature female gametophyte, gametes, and globular embryo proper. In addition, pollen and sperm group separately from the other samples analyzed. Hierarchical clustering of genes/samples was based on euclidean distance and hierarchical agglomerative clustering. Colors are scaled per row and yellow denotes high expression and blue low expression. (TIF) [file pbio.1001155.s009.tif]

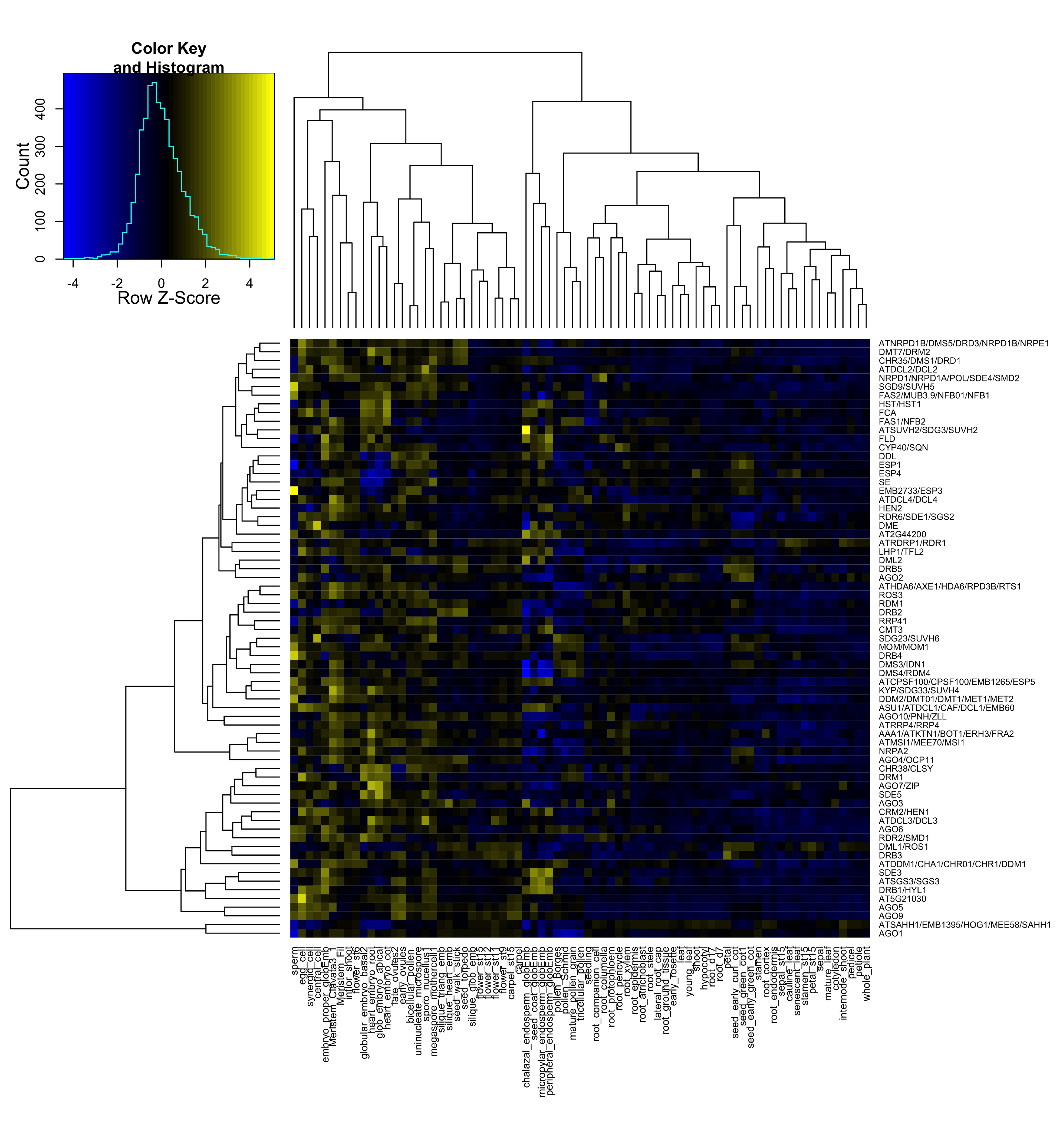

Supplement: Figure S10 — Expression of genes involved in DNA methylation and small RNA pathways across the tissue atlas. Heatmap of log2 transformed expression values for 69 genes involved in DNA methylation and different small RNA pathways (see Figure S9) of all samples composing the tissue atlas of plus additional samples of male gametogenesis [92]. The datasets of mature pollen cluster closer to a variety of sporophytic tissue samples and to endosperm and seed coat, and group separately from different reproductive tissues and cell types, including flowers, siliques, seeds, embryo, ovules, samples from megasporogenesis, cells composing the female gametophyte, and sperm, but also meristems, carpels, and inflor_shoot. Within this subgroup, sperm is distinct from the other samples. Hierarchical clustering of genes/samples was based on euclidean distance and hierarchical agglomerative clustering. Colors are scaled per row and yellow denotes high expression and blue low expression. (TIF) [file pbio.1001155.s010.tif]
