# Supplementary material for: Transcriptome Analysis of the Arabidopsis Megaspore Mother Cell Uncovers the Importance of RNA Helicases for Plant Germline Development
Source: PLoS Biol. 2011 Sep 20;9(9):e1001155. doi: 10.1371/journal.pbio.1001155 (PMC3176755; doi:10.1371/journal.pbio.1001155)
Supplement: Table S2 — Evidence of expression of genes selected for data validation (Figure 2). AtPANP present and absent calls and mean expression values as calculated by dChip [81]. (DOC) [file pbio.1001155.s012.doc]

**Table S2:**

| **AGI** | **P/A calls AtPANP MMC** | **P/A calls AtPANP sporo_nucllus** | **higher mean expression in** | **MEAN expression levels in MMC** | **MEAN expression levels in sporo_nucellus** |
| --- | --- | --- | --- | --- | --- |
| *AT3G14700* | P | P | MMC | 1485.8 | 908.2 |
| *AT2G30940* | P | P | MMC | 2310.3 | 1449.3 |
| *AT1G31150* | P | P | MMC | 1051.0 | 488.2 |
| *AT2G29210* | P | P | MMC | 2034.7 | 1063.0 |
| *AT1G72320* | P | P | MMC | 1198.0 | 519.0 |
| *AT5G23080* | P | P | MMC | 1024.2 | 528.7 |
| *AT1G11270* | P | P | MMC | 588.7 | 299.1 |
| *AT3g19510* | P | P | MMC | 541.4 | 305.0 |
| *AT3g21175* | P | P | MMC | 630.8 | 252.2 |
| *AT2G24500* | P | P | MMC | 1762.1 | 1192.0 |
| *AT1G31240* | P | A | MMC | 375.0 | 120.8 |
| *AT1G80440* | P | P | MMC | 188.7 | 104.0 |
| *PUM12* | M | P | sporo_nucellus | 628.1 | 863.8 |
| *ABCB19* | M | P | sporo_nucellus | 448.3 | 637.5 |
