# Supplementary material for: Transcriptome Analysis of the Arabidopsis Megaspore Mother Cell Uncovers the Importance of RNA Helicases for Plant Germline Development
Source: PLoS Biol. 2011 Sep 20;9(9):e1001155. doi: 10.1371/journal.pbio.1001155 (PMC3176755; doi:10.1371/journal.pbio.1001155)
Supplement: Table S3 — Gene ontology analysis. Analysis of molecular functions upregulated based on the 796 genes enriched in the MMC transcriptome as compared to the transcriptomes of egg cell, central cell, and synergids (p value <0.01). (DOC) [file pbio.1001155.s013.doc]

Table S3:

| **GO.ID** | **term** | **significant** | **expected** | **p-value** |
| --- | --- | --- | --- | --- |
| GO:0003735 | structural constituent of ribosome | 77 | 11.7 | < 1e-30 |
| GO:0008135 | translation factor activity, nucleic acid binding | 20 | 4.11 | 5.50E-09 |
| GO:0003723 | RNA binding | 38 | 14.15 | 1.50E-06 |
| GO:0015450 | P-P-bond-hydrolysis-driven protein transmembrane transporter activity | 8 | 1.86 | 0.0005 |
| GO:0030527 | structural constituent of chromatin | 3 | 0.2 | 0.0006 |
| GO:0003989 | acetyl-CoA carboxylase activity | 3 | 0.2 | 0.0006 |
| GO:0016004 | phospholipase activator activity | 3 | 0.24 | 0.0011 |
| GO:0003755 | peptidyl-prolyl cis-trans isomerase activity | 8 | 2.17 | 0.0014 |
| GO:0045153 | electron transporter, transferring electrons within coQH2-cytochrome c reductase activity complex | 2 | 0.08 | 0.0016 |
| GO:0004013 | adenosylhomocysteinase activity | 2 | 0.08 | 0.0015 |
| GO:0005507 | copper ion binding | 13 | 4.47 | 0.0015 |
| GO:0008308 | voltage-gated anion channel activity | 4 | 0.59 | 0.0023 |
| GO:0008186 | RNA-dependent ATPase activity | 3 | 0.32 | 0.0030 |
| GO:0008026 | ATP-dependent helicase activity | 9 | 3.08 | 0.0036 |
| GO:0042277 | peptide binding | 4 | 0.67 | 0.0038 |
| GO:0004784 | superoxide dismutase activity | 3 | 0.36 | 0.0043 |
| GO:0048040 | UDP-glucuronate decarboxylase activity | 2 | 0.12 | 0.0046 |
| GO:0008097 | 5s rRNA binding | 2 | 0.12 | 0.0046 |
| GO:0004634 | phosphopyruvate hydratase activity | 2 | 0.12 | 0.0046 |
| GO:0008705 | methionine synthase activity | 2 | 0.12 | 0.0046 |
| GO:0003871 | 5-methyltetrahydropteroyltriglutamate-homocysteine S-methyltransfase activity | 2 | 0.12 | 0.0046 |
| GO:0017163 | basal transcription repressor activity | 2 | 0.12 | 0.0046 |
| GO:0004066 | asparagine synthase (glutamine-hydrolyzing) activity | 2 | 0.12 | 0.0046 |
| GO:0004743 | pyruvate kinase activity | 3 | 0.40 | 0.0060 |
| GO:0004478 | methionine adenosyltransferase activity | 2 | 0.16 | 0.0089 |
| GO:0019202 | amino acid kinase activity | 2 | 0.16 | 0.0089 |
| GO:0017077 | oxidative phosphorylation uncoupler activity | 2 | 0.16 | 0.0089 |
| GO:0050661 | NADP or NADPH binding | 2 | 0.16 | 0.0089 |
