# Supplementary material for: Transcriptome Analysis of the Arabidopsis Megaspore Mother Cell Uncovers the Importance of RNA Helicases for Plant Germline Development
Source: PLoS Biol. 2011 Sep 20;9(9):e1001155. doi: 10.1371/journal.pbio.1001155 (PMC3176755; doi:10.1371/journal.pbio.1001155)
Supplement: Table S4 — Enrichment of PFAM-domains analyzed for genes with preferential MMC expression. Enrichment of protein families was tested by a two-sided Fischer exact test. P values <0.01 for genes with significantly higher expression in the MMC in each contrast as compared to the cells of the mature female gametophyte (egg cell, central cell, synergids) were considered significant. (DOC) [file pbio.1001155.s014.doc]

**Table S4:**

| **ID** | **Significant** | **Expected** | **p-value** | **annotation** |
| --- | --- | --- | --- | --- |
| PF00076 | 22 | 7.59 | 2.99E-05 | RNA recognition motif |
| PF00080 | 3 | 0.15 | 0.0014 | Copper /Zink superoxide dismutase |
| PF00118 | 6 | 0.81 | 0.0004 | TCP-1/cpn60 chaperonin family |
| PF00153 | 7 | 2.11 | 0.0008 | Mitochondrial carrier protein |
| PF00163 | 2 | 0.74 | 0.0073 | Ribosomal protein S4/S9 N-terminal domain |
| PF00224 | 3 | 0.37 | 0.0099 | Pyruvate kinase, barrel domain |
| PF00227 | 5 | 0.70 | 0.0014 | Proteasome subunit |
| PF00237 | 2 | 0.07 | 0.0073 | Ribosomal protein L22p/L17e |
| PF00270 | 8 | 2.70 | 0.0086 | DEAD/DEAH box helicase |
| PF00298 | 3 | 0.22 | 0.0032 | Ribosomal protein L11, RNA binding domain |
| PF00428 | 6 | 0.52 | 5.31E-05 | 60S acidic ribosomal protein |
| PF00466 | 4 | 0.22 | 0.0002 | Ribosomal protein L11 |
| PF00467 | 3 | 0.26 | 0,0045 | KOW motif |
| PF00505 | 4 | 0.52 | 0.0034 | HMG (high mobility group) box |
| PF00572 | 3 | 0.22 | 0.0033 | Ribosomal protein L13 |
| PF00658 | 3 | 0.26 | 0.0045 | Poly-adenlyate binding protein, unique domain |
| PF00670 | 2 | 0.07 | 0.0073 | S-adenosyl-L-homocysteine hydrolase, NAD binding domain |
| PF00687 | 3 | 0.18 | 0.0022 | Ribosomal L1p/L10e family |
| PF00917 | 5 | 1.03 | 0.0060 | MATH domain |
| PF01092 | 2 | 0.07 | 0.0073 | Ribosomal protein S6e |
| PF01201 | 3 | 0.11 | 0.0008 | Ribosomal protein S8e |
| PF01459 | 4 | 0.30 | 0.0006 | Eukaryotic porin |
| PF01479 | 4 | 0.48 | 0.0027 | S4 domain |
| PF01798 | 3 | 0.11 | 0.0008 | Putative snoRNA binding domain |
| PF01912 | 2 | 0.74 | 0.0073 | eIF-6 family |
| PF01918 | 4 | 0.22 | 0.0003 | Alba |
| PF02167 | 2 | 0.07 | 0.0073 | Cytochrome C1 family |
| **ID** | **Significant** | **Expected** | **p-value** | **annotation** |
| PF02466 | 4 | 0.52 | 0.0034 | Tim17 / Tim22 / Tim23 family |
| PF02887 | 3 | 0.33 | 0.0080 | Thiolase C-terminal domain |
| PF03946 | 3 | 0.19 | 0.0022 | Ribosomal protein L11, N-terminal domain |
| PF05221 | 2 | 0.07 | 0.0073 | S-adenosyl-L-homocysteine hydrolase |
| PF08060 | 3 | 0.15 | 0.0014 | NOSIC (NUC001) domain |
| PF08069 | 2 | 0.07 | 0.0073 | Ribosomal S13/S15 N-terminal domain |
| PF08156 | 3 | 0.11 | 0.0010 | NOP5NT (NUC127) domain |
