# Supplementary material for: Transcriptome Analysis of the Arabidopsis Megaspore Mother Cell Uncovers the Importance of RNA Helicases for Plant Germline Development
Source: PLoS Biol. 2011 Sep 20;9(9):e1001155. doi: 10.1371/journal.pbio.1001155 (PMC3176755; doi:10.1371/journal.pbio.1001155)
Supplement: Table S6 — Gene ontology analysis. Analysis of molecular functions and biological process upregulated based on the 134 genes enriched in the sporo_nucellus transcriptome as compared to the transcriptomes of the tissue atlas not including the MMC (p value <0.01). (DOC) [file pbio.1001155.s016.doc]

Table S6:

| **Biological process** |  |  |  |  |  |
| --- | --- | --- | --- | --- | --- |
| **GO.ID** | **Term** | **Annotated** | **Significant** | **Expected** | **p-value** |
| GO:0006857 | oligopeptide transport | 59 | 4 | 0.37 | 0.0005 |
| **Molecular function** |  |  |  |  |  |
| **GO.ID** | **Term** | **Annotated** | **Significant** | **Expected** | **p-value** |
| GO:0003993 | acid phosphatase activity | 36 | 4 | 0.24 | 9e-05 |
| GO:0004722 | protein serine/threonine phospatase activity | 134 | 5 | 0.89 | 0.0020 |
| GO:0003735 | structural constituent of ribosome | 296 | 7 | 1.96 | 0.0035 |
| GO:0003723 | RNA binding | 358 | 7 | 2.37 | 0.0097 |
