# Supplementary material for: Transcriptome Analysis of the Arabidopsis Megaspore Mother Cell Uncovers the Importance of RNA Helicases for Plant Germline Development
Source: PLoS Biol. 2011 Sep 20;9(9):e1001155. doi: 10.1371/journal.pbio.1001155 (PMC3176755; doi:10.1371/journal.pbio.1001155)
Supplement: Table S8 — Gene ontology analysis of MMC enriched genes. Gene ontology analysis to identify biological processes and molecular functions upregulated in 82 genes enriched in the MMC transcriptome as compared to the tissue atlas (sporo_nucellus sample excluded, adjusted p value in each contrast <0.01). (DOC) [file pbio.1001155.s018.doc]

**Table S8:**

| **Biological process** |  |  |  |  |  |
| --- | --- | --- | --- | --- | --- |
| **GO.ID** | **Term** | **Annotated** | **Significant** | **Expected** | **p-value** |
| GO:0006571 | tyrosine biosynthetic process | 2 | 1 | 0.01 | 0.0076 |
| GO:0006412 | translation | 459 | 6 | 1.75 | 0.0079 |
| **Molecular function** |  |  |  |  |  |
| **GO.ID** | **Term** | **Annotated** | **Significant** | **Expected** | **p-value** |
| GO:0003993 | acid phosphatase activity | 36 | 3 | 0.15 | 0.0004 |
| GO:0008026 | ATP-dependent helicase activity | 78 | 3 | 0.32 | 0.0042 |
| GO:0004665 | prephenate dehydrogenase (NADP+) activity | 2 | 1 | 0.01 | 0.0083 |
| GO:0003701 | RNA polymerase I transcription factor activity | 2 | 1 | 0.01 | 0.0083 |
