# Supplementary material for: Transcriptome Analysis of the Arabidopsis Megaspore Mother Cell Uncovers the Importance of RNA Helicases for Plant Germline Development
Source: PLoS Biol. 2011 Sep 20;9(9):e1001155. doi: 10.1371/journal.pbio.1001155 (PMC3176755; doi:10.1371/journal.pbio.1001155)
Supplement: Table S10 — Primers for cloning. List of primers used for cloning of in situ probes and promoter-GUS expression constructs. (DOC) [file pbio.1001155.s020.doc]

**Table S10:**

| **AGI** | **construct** | **forward primer** | **reverse primer** |
| --- | --- | --- | --- |
| *AT3G14700* | *in situ* | 5’-CTTGCTTCGAGAGTGGAAGC-3’ | 5’-ACACCAACAACCTTGCCTTC-3’ |
| *AT2G30940* | *in situ* | 5’-GATCCTCATCACAGCTTCTGC-3’ | 5’-GATTCCGCGATACACCGTTG-3’ |
| *AT1G31150* | *in situ* | 5’-ATGCTCACATTCAGGTAAGC-3’ | 5’-GTAACATCTCCAACACATCTCC-3’ |
| *AT2G29210* | *in situ* | 5’-GATCCAGATCACGTTCCGTG-3’ | 5’-TAGCAGGTGGTGATGGTGAC-3’ |
| *AT1G72320* | *in situ* | 5’-GAAAGAGATTGATCCAGAGACTTC-3’ | 5’-AAGTAGAGTTTGCAAGACATGAC-3’ |
| *AT5G23080* | *in situ* | 5’-CTAAGCCAACTGTTTCTGCTC-3’ | 5’-TAGTAGTCATGACCGTTACCTTC-3’ |
| *AT1G11270* | GUS-expression  construct | 5’-GGGGACAAGTTTGTACAAAAAAGCAGGCTTTGGTCTACTCTGCATCCAC-3’ | 5’-GGGGACCACTTTGTACAAGAAAGCTGGGTCATTGCTTCTTCTTGTCTCTCTATTCTCTAATTG-3’ |
| *AT3G19510* | GUS-expression  construct | 5’-GGGGACAAGTTGTACAAAAAAGCACGGCTGGCATTCCTAGAGAGATCGCAG-3’ | 5’-GGGACCACTTTGTACAAGAAAGCTGGGTCATCTATCCTCGAAGATATGCACTC-3’ |
| *AT3G21175* | GUS-expression  construct | 5’-GGGGACAAGTTTGTACAAAAAAGCAGGCTCAACATGGTATTTAGTTCTTGG-3’ | 5’-GGGGACCACTTTGTACAAGAAAGCTGGGTCTGCAATTTGTTACCAATTACATC-3’ |
| *AT2G24500* | GUS-expression  construct | 5’-GGGGACAAGTTTGTACAAAAAAGCAGGCTCATTGTCTTTCTCGTTGTGC-3’ | 5’-GGGGACCACTTTGTACAAGAAAGCTGGGTGAGGATTAGGGATGGCTTTGG-3’ |
